# The SUMOylation pathway suppresses arbovirus replication in *Aedes aegypti* cells

**DOI:** 10.1371/journal.ppat.1009134

**Published:** 2020-12-22

**Authors:** Samuel Stokes, Floriane Almire, Michael H. Tatham, Steven McFarlane, Peter Mertens, Emilie Pondeville, Chris Boutell

**Affiliations:** 1 MRC-University of Glasgow Centre for Virus Research, Glasgow, Scotland, United Kingdom; 2 The Pirbright Institute, Pirbright, Woking, England, United Kingdom; 3 Centre for Gene Regulation and Expression, School of Life Sciences, University of Dundee, Dundee, Scotland, United Kingdom; University of California Davis, UNITED STATES

## Abstract

Mosquitoes are responsible for the transmission of many clinically important arboviruses that cause significant levels of annual mortality and socioeconomic health burden worldwide. Deciphering the mechanisms by which mosquitoes modulate arbovirus infection is crucial to understand how viral-host interactions promote vector transmission and human disease. SUMOylation is a post-translational modification that leads to the covalent attachment of the Small Ubiquitin-like MOdifier (SUMO) protein to host factors, which in turn can modulate their stability, interaction networks, sub-cellular localisation, and biochemical function. While the SUMOylation pathway is known to play a key role in the regulation of host immune defences to virus infection in humans, the importance of this pathway during arbovirus infection in mosquito vectors, such as *Aedes aegypti* (*Ae*. *aegypti*), remains unknown. Here we characterise the sequence, structure, biochemical properties, and tissue-specific expression profiles of component proteins of the *Ae*. *aegypti* SUMOylation pathway. We demonstrate significant biochemical differences between *Ae*. *aegypti* and *Homo sapiens* SUMOylation pathways and identify cell-type specific patterns of SUMO expression in *Ae*. *aegypti* tissues known to support arbovirus replication. Importantly, depletion of core SUMOylation effector proteins (SUMO, Ubc9 and PIAS) in *Ae*. *aegypti* cells led to enhanced levels of arbovirus replication from three different families; Zika (*Flaviviridae*), Semliki Forest (*Togaviridae*), and Bunyamwera (*Bunyaviridae*) viruses. Our findings identify an important role for mosquito SUMOylation in the cellular restriction of arboviruses that may directly influence vector competence and transmission of clinically important arboviruses.

## Author summary

Half the world’s population is at risk of infection from arboviruses transmitted by mosquitoes. Deciphering the viral-host interactions that influence the outcome of arbovirus infection in mosquitoes is beneficial to the development of future vector control strategies to limit arbovirus transmission and viral emergence within the human population. Similar to humans, mosquitoes possess different immune pathways to limit the replication of arboviruses. While the Small Ubiquitin-like MOdifier (SUMO) pathway is known to play an important role in the regulation of immune defences to viral infection in humans, the influence of this pathway during arbovirus infection in mosquito cells is currently unknown. Here we define the conservation, biochemical activity, and tissue distribution of the core effector proteins of the *Aedes aegypti* SUMOylation pathway. We show that the mosquito SUMOylation pathway plays a broadly antiviral role against a wide range of clinically important arboviruses, including Zika, Semliki Forest, and Bunyamwera viruses. Our findings identify SUMOylation as an important component of the antiviral response to arbovirus infection in mosquito cells.

## Introduction

Many clinically important arthropod-borne viruses (arboviruses), such as dengue (DENV), Zika (ZIKV) (Flaviviruses; *Flaviviridae*), chikungunya (CHIKV) (Alphavirus; *Togaviridae*) and Rift Valley Fever (Phlebovirus; *Bunyaviridae*) viruses, are transmitted by the mosquito vector *Aedes aegypti* (*Ae*. *aegypti*, *Aa*). *Aedes*-borne pathogens represent a substantial worldwide public health burden due to an ever expanding geographical vector range and associated threat of viral emergence and epidemic disease [1–4]. For example, approximately half of the world’s population is now estimated to be at risk from DENV with around 390 million new infections worldwide every year [5]. In addition, arboviral diseases transmitted by *Aedes* have an important socioeconomic impact, with the 2015–2016 Zika outbreak in Latin America and the Caribbean estimated to cost 18 billion USD [6]. Without efficient treatments or vaccines against the majority of arbovirus infections, vector control is the most widely utilized strategy to limit viral transmission [7]. Insecticides have been commonly used as the first line of defence, but increased mosquito resistance hampers their efficacy [8,9]. As such, there is a need to develop new and effective vector control measures [10]. In this context, it is crucial to improve our understanding of mosquito biology and mosquito-arbovirus interactions to identify targets that influence arbovirus infection directly in mosquitoes.

The Small Ubiquitin-like MOdifier (SUMO) protein covalently modifies target proteins (in a process termed SUMOylation) in a reversible manner. This post-translational modification regulates a wide variety of cellular processes, including the cell cycle, transcription, DNA repair, protein stability and sub-cellular localization [11]. In *Homo sapiens* (*H*. *sapiens)*, the process of SUMOylation is conducted in a 3-step enzymatic cascade, involving the E1 SUMO activating enzyme complex SAE1/2, the E2 conjugating enzyme Ubc9 (UBE2I), and E3 SUMO ligases, including the Protein Inhibitor of Activated STAT (PIAS) family of proteins [11]. *H*. *sapiens* possess 5 SUMO homologues, of which only SUMO1, 2, and 3 are constitutively expressed and conjugated to target proteins [12]. SUMO modification typically occurs on lysine (Lys/K) residues within a SUMO Consensus Motif (SCM) Ψ-Lys-X-[Asp/Glu] of a target protein, where Ψ represents a hydrophobic amino acid and X represents any amino acid [13]. SUMO2 and SUMO3 both contain an internal SCM that readily supports poly-SUMO2/3 chain formation [14,15]. SUMO chains have many important cellular functions, including roles in meiosis, mitosis, DNA repair, and proteasomal degradation [16]. In contrast, SUMO1 does not possess an internal SCM, and is therefore generally considered to be conjugated as a monomer onto target proteins or incorporated into poly-SUMO chains as a chain terminator [14].

SUMOylation plays an important function, both positively and negatively, in the replication of a wide range of DNA and RNA viruses in mammalian cells, either directly through the SUMOylation of viral proteins or indirectly by regulation of immune defences [17–19]. To date, studies to determine the role of SUMOylation during arbovirus infection have focused on flaviviruses in mammalian cells [20–23]. While two of these studies demonstrated depletion of Ubc9 could restrict DENV replication [20,21], a third study showed that SUMOylation of the DENV non-structural 5 protein (NS5) decreased ubiquitin-mediated degradation of NS5 to promote infection [23]. This proviral role for host SUMOylation in mammalian cells was supported by recent findings from Zhu *et al*. [22], which demonstrated the SUMOylation inhibitor 2-D08 to decrease flavivirus (ZIKV, DENV, Yellow fever virus, West Nile virus, and Japenese Encephalitis virus) RNA levels during infection. However, it remains to be determined what role (proviral or antiviral) SUMOylation plays during arbovirus infection in mosquito cells.

Genes of the SUMOylation pathway are conserved in various arthropods, including insects [24,25]. Most of the knowledge on SUMO function in insects has been gained from studies in the fruit fly *Drosophila melanogaster* (*D*. *melanogaster)*. In this species, SUMO modification is known to be involved in embryogenesis, metamorphosis, gut regeneration, and innate immunity [26]. However, the functional conservation of the different effectors involved in the SUMOylation pathway, along with its role during infection with alphaviruses, flaviviruses, and bunyaviruses in arthropods has yet to be determined.

Here we characterise the sequence homology and functional conservation of the SUMOylation pathway between *Ae*. *aegypti* and *H*. *sapiens*. Our analysis reveals that *Aa*SUMO is functionally more comparable to *Hs*SUMO1 than *Hs*SUMO3, the closest related orthologue, due to the lack of an internal SCM. Consistent with SUMOylation in *H*. *sapiens*, we show that the sole *Ae*. *aegypti* PIAS (*Aa*PIAS) SUMO E3 ligase stimulates *Aa*SUMO poly-SUMO chain formation in a SIZ/PIAS (SP) RING-domain dependent manner. We identify differential patterns of SUMO expression in *Ae*. *aegypti* cells and tissues known to support arbovirus replication. Finally, we present compelling evidence for a broad antiviral effect of *Ae*. *aegypti* host SUMOylation against arboviruses. Depletion of *Aa*SUMO, *Aa*Ubc9, or *Aa*PIAS resulted in increased arbovirus replication from three independent families; bunyamwera virus (BUNV), Semliki Forest virus (SFV) and ZIKV. To our knowledge, this research is the first study to investigate the biochemical properties and biological significance of the SUMOylation pathway on arbovirus replication in *Ae*. *aegypti* cells. Our findings reveal an important antiviral role for *Aa*SUMOylation in the regulation of antiviral defences to arbovirus infection in mosquito cells.

## Results

### *Ae*. *aegypti* and *H*. *sapiens* SUMOylation pathways are highly conserved

Bioinformatic studies had previously identified two *Ae*. *aegypti* SUMO orthologue genes (AAEL015064; AAEL013787), along with gene orthologues of SAE1 and SAE2 (AAEL000091 and AAEL010641, respectively), Ubc9 (AAEL007477), and PIAS (AAEL015099) [24,25]. Following the recent re-sequencing and updated annotation of the *Ae*. *aegypti* genome (AaegL5.1, available at https://www.vectorbase.org/; [27]), the gene ID of Ubc9 has changed to AAEL027903. In addition, our analysis identified only one SUMO gene (AAEL015064), with sequences relating to the second putative gene (AAEL013787) being re-annotated to that of a calcium ion transporter gene (AAEL019513). Mass spectrometry analysis of *Ae*. *aegypti* AF5 cells identified peptides unique to each protein of the *Ae*. *aegypti* SUMOylation pathway (SUMO [AAEL015064], SAE1/2, Ubc9, and PIAS), with the exception of the putative AAEL013787 SUMO protein (S1 File). Therefore, AAEL015064 is the only SUMO gene product abundantly expressed in *Ae*. *aegypti* AF5 cells (hereafter referred to as *Aa*SUMO).

**Fig 1:**
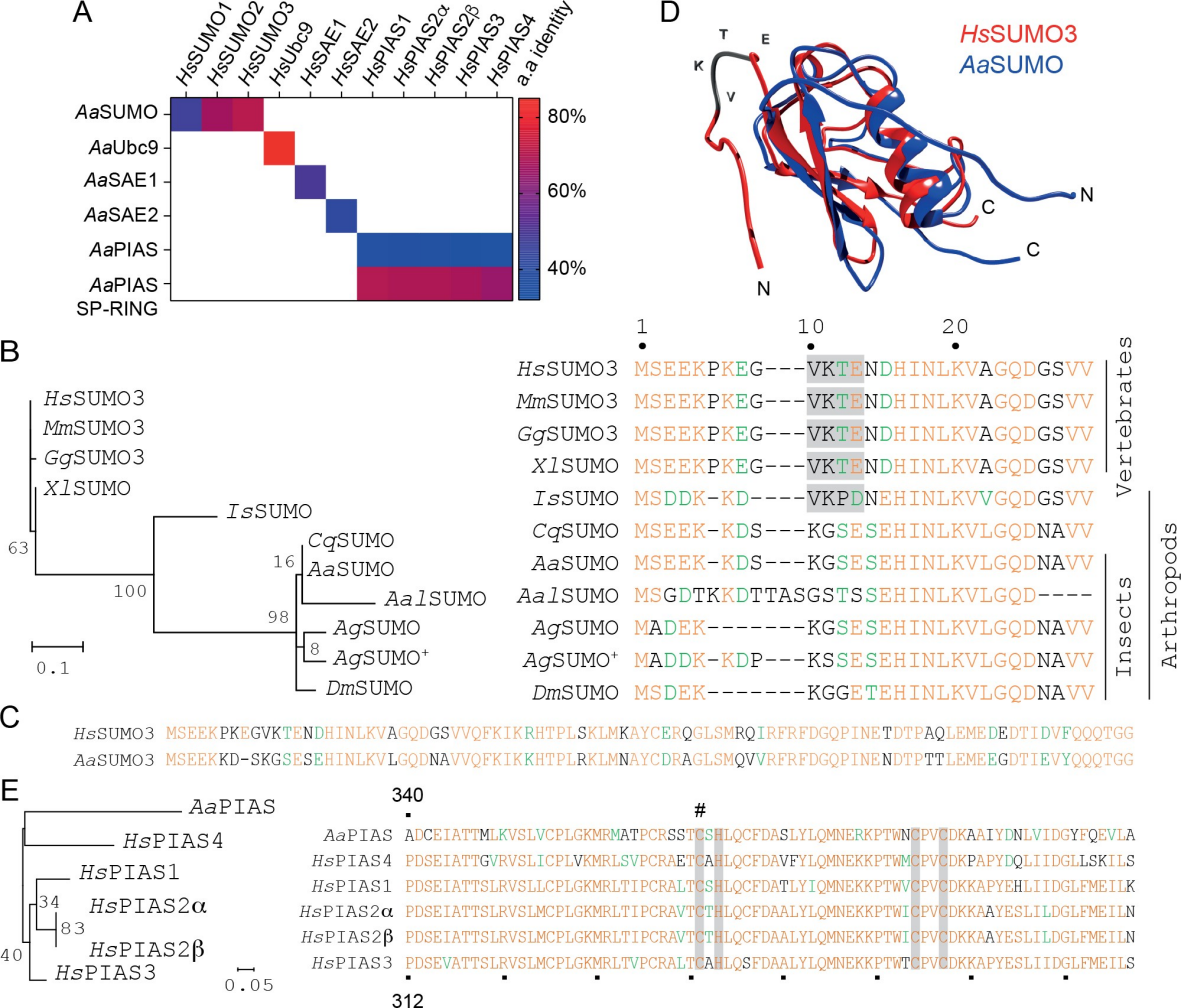
The *Aedes aegypti* SUMOylation pathway is highly conserved. (A) Colour-coded pairwise identity matrix showing the percentage amino acid (a.a) identity between orthologue proteins of the *Aedes aegypti* and *Homo sapiens* SUMOylation pathways. Each coloured cell represents the percentage identity score between two sequences. Colour key showing the correspondence between percentage identity and the colours displayed shown. (B) Phylogenetic tree and N-terminal amino acid alignment of SUMO proteins from a selection of model organisms (SUMO Uniprot accession number). *Hs*, *Homo sapiens* (P55854); *Mm*, *Mus musculus* (Q9Z172); *Gg*, *Gallus gallus* (Q5ZHQ1); *Xl*, *Xenopus laevis* (Q6DK72); *Is*, *Ixodes scapularis* (B7PKS5); *Cq*, *Culex quinquefasciatus* (B0WVZ6); *Aa*, *Aedes aegypti* (Q16EQ3); *Aal*, *Aedes albopictus* (A0A182H3N0); *Ag*, *Anopheles gambiae* (Q7PNJ2 and Q7PY16 ^**+**^); *Dm*, *Drosophila melanogaster* (O97102). Branch lengths are proportional to sequence divergence with bootstrap values shown. Conserved residues are shown in orange; residues with similarity in green. SUMO consensus motifs (SCM; Ψ-Lys(K)-X-Asp(D)/Glu(E), where Ψ represents a branched hydrophobic amino acid and X represents any amino acid) are higlighted in grey. (C) Amino acid alignment of full length *Aa*SUMO plotted against *Hs*SUMO3. (D) Predicted structure of *Aa*SUMO (blue) plotted against the resolved structure of *Hs*SUMO3 (red; PDB ID: 2MP2; [85]). Position and sequence of SCM in *Hs*SUMO3 highlighted (VKTE; grey). (E) Phylogenetic tree and amino acid alignment of the catalytic SP-RING domain of orthologue PIAS proteins from *Ae*. *aegypti* and *H*. *sapiens*. Uniprot accession numbers; *Aa*PIAS (Q1DH55), *Hs*PIAS1 (O75925), *Hs*PIAS2α (O75928-2), *Hs*PIAS2β (O75928-1), *Hs*PIAS3 (Q9Y6X2), and *Hs*PIAS4 (Q8N2W9). Branch lengths proportional to sequence divergence with bootstrap values shown. Residues of *Aa*PIAS 340–418, *Hs*PIAS4 311–388, *Hs*PIAS3 312–390, *Hs*PIAS2α 331–409, *Hs*PIAS2β 331–409, and *Hs*PIAS1 320–398 shown. Zn^**2+**^ binding residues highlighted by grey bars; cysteine (C371) marked with #.

Alignment of SUMOylation effector proteins (SUMO, SAE1/2, Ubc9, and PIAS) between *Ae*. *aegypti* and *H*. *sapiens* demonstrated a high degree of protein conservation. *Aa*SAE1, *Aa*SAE2, and *Aa*Ubc9 shared 42%, 50%, and 85% amino acid identity with their respective *H*. *sapiens* orthologues (Fig 1A). Homology between *Aa*SUMO and *Hs*SUMO1-3 proteins ranged from 47 to 71% identity, with *Aa*SUMO showing the highest degree of similarity to that of *Hs*SUMO3 (Fig 1A). Phylogenetic alignment of SUMO proteins from different species indicated that SUMO orthologues from *Ae*. *aegypti* and other mosquito species lack an N-terminal SCM, contrary to the chelicerate *Ixodes scapularis* and vertebrate SUMO orthologues (Fig 1B and 1C; [25]). Homology modelling confirmed *Aa*SUMO to contain a classical ubiquitin-related modifier tertiary structure with flexible N- and C-terminal regions (Fig 1D; [25,28]). In *H*. *sapiens*, PIAS SUMO E3 ligases are known to increase the rate of SUMO modification and poly-SUMO chain formation [29,30]. In contrast to the five PIAS proteins expressed by *H*. *sapiens*, only one PIAS protein has been identified in *Ae*. *aegypti* [24]. The amino acid identity of *Aa*PIAS ranged from 32 to 37% between *Hs*PIAS orthologues, increasing up to 70% in the catalytic SP-RING domain (Fig 1A). Sequence alignment confirmed the presence of Zn^2+^-coordinating residues within the SP-RING required for SUMO ligase activity (Fig 1E; grey bars) [31]. These data indicate that the function of the SP-RING domain is likely to be conserved between *H*. *sapiens* and *Ae*. *aegypti*. Overall, our analysis revealed a high degree of protein conservation between *Ae*. *aegypti* and *H*. *sapiens* SUMOylation pathways. These data suggest a functional conservation of this pathway between mosquitoes and humans, with the notable exception of an absent N-terminal SCM within *Aa*SUMO.

### *Aa*PIAS stimulates the efficient formation of poly-*Aa*SUMO chains

**Fig 2:**
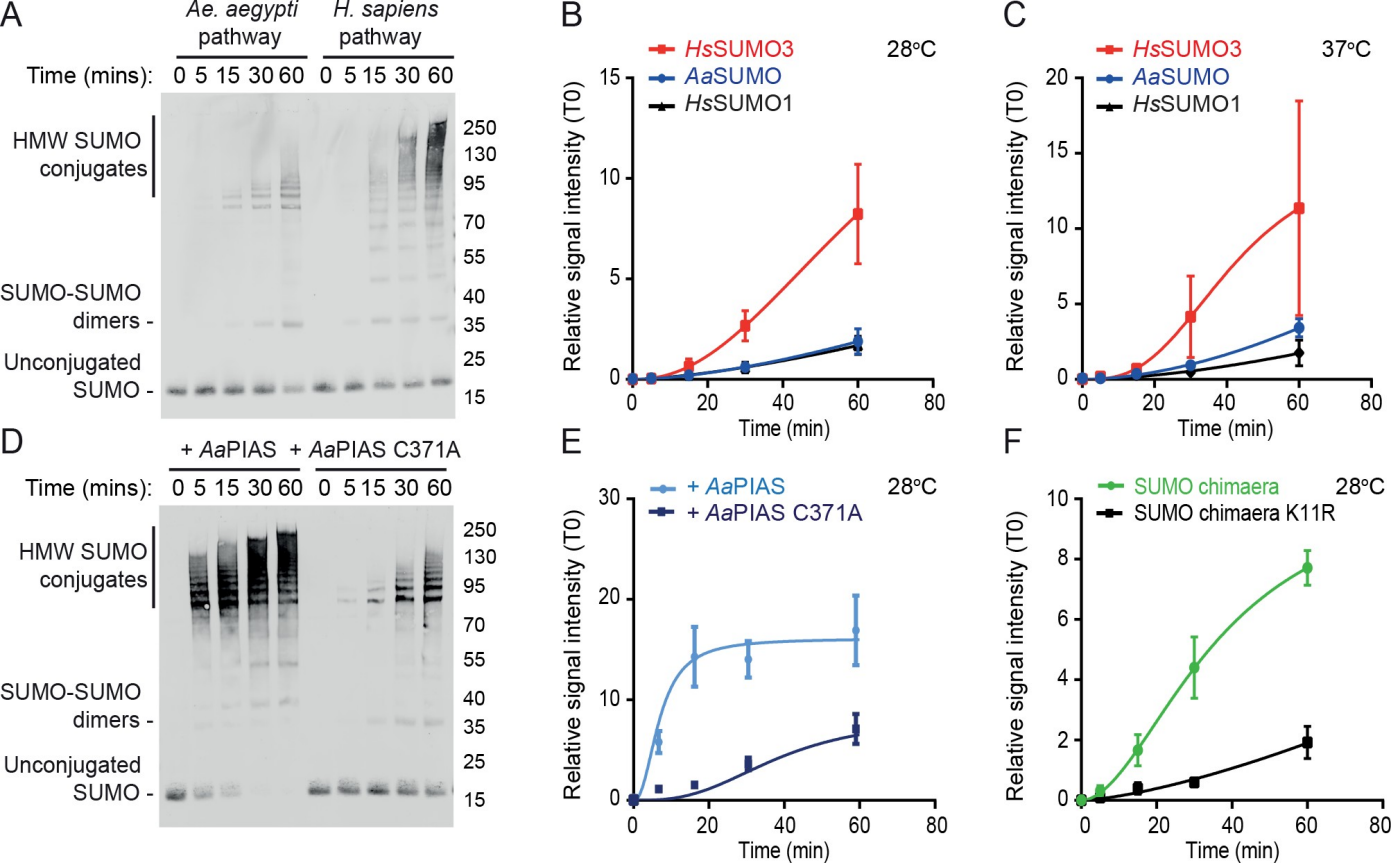
*Aa*PIAS is required for efficient poly-*Aa*SUMO chain formation. Purified *Ae*. *aegypti* and *H*. *sapiens* SUMO, Ubc9, and SAE1/2 recombinant proteins (50 ng each per reaction, S1 Fig) were incubated in the presence of 5 mM ATP for the indicated time points (minutes, min). (A) Representative western blot showing the accumulation of high molecular weight (HMW) SUMO conjugates over time at 28°C. Free SUMO, SUMO-SUMO dimers, and HMW conjugates highlighted. Molecular mass markers shown. (B/C) Quantitation of the accumulation of HMW SUMO conjugates (as in A; ≥ SUMO dimers) at 28 or 37°C (B and C, respectively). N≥3 independent reactions per condition; values normalized to T = 0 min (T0); mean and standard error of mean plotted. (D/E) *Ae*. *aegypti* SUMOylation pathway enzymes were incubated in the presence of WT or catalytically inactive (C371A) *Aa*PIAS (10 ng) for the indicated time points at 28°C. (D) Representative western blot showing the accumulation of HMW SUMO conjugates in the presence of WT *Aa*PIAS. (E) Quantitation of the accumulation of HMW SUMO conjugates (as in D and described in B). (F) *Ae*. *aegypti* SUMOylation pathway enzymes (SAE1/2 and Ubc9) were incubated in the presence of chimaeric SUMO (amino acids 1–14 of *Hs*SUMO3 [including WT or mutant (K11R) SCM] in frame with the C-terminus of *Aa*SUMO) for the indicate time points at 28°C. Western blots were quantified for the accumulation of HMW SUMO conjugates (as described in B).

As *Aa*SUMO lacks an N-terminal SCM, this protein may not readily support poly-SUMO chain formation, unlike its closest *H*. *sapiens* orthologue *Hs*SUMO3 [14]. We tested this hypothesis by incubating together recombinant proteins (S1 Fig) of either the *Aa*SUMOylation pathway (*Aa*SUMO, *Aa*Ubc9, and *Aa*SAE1/2) or the *Hs*SUMOylation pathway (*Hs*SUMO3, *Hs*Ubc9, and *Hs*SAE1/2) at either 28 or 37°C, the optimal temperatures for mosquito and mammalian cells, respectively. We detected a build-up of *Aa*SUMO-dimers along with high molecular weight (HMW) SUMO conjugates indicative of SAE1/2 auto-SUMOylation (Fig 2A; [32,33]). The formation of HMW *Aa*SUMO conjugates was more comparable to *Hs*SUMO1 than *Hs*SUMO3 at either 28 or 37°C (Fig 2B and 2C). These data indicate that *Aa*SUMO is more biochemically similar to that of *Hs*SUMO1 than *Hs*SUMO3 with respect to supporting the formation of HWM SUMO protein conjugates, consistent with the lack of an internal SCM (Fig 1B; [25]). In order to determine the effect of *Aa*PIAS on poly-*Aa*SUMO chain formation, biochemical assays were repeated in the presence of wild-type (WT) or catalytically inactive (C371A; Fig 1E) *Aa*PIAS. Addition of WT *Aa*PIAS led to a rapid build-up of HMW SUMO conjugates relative to *Aa*PIAS C371A (Fig 2D and 2E). Collectively, these data indicate that *Aa*PIAS stimulates the efficient formation of HMW SUMO conjugates in an SP-RING dependent manner. We next sought to investigate why *Aa*SUMO could not efficiently form chains in the absence of *Aa*PIAS. We hypothesized that this was due to the absence of an SCM (Fig 1B; [25]). We generated a chimeric SUMO protein, in which the N-terminus of *Aa*SUMO (amino acids 1–13) was replaced by the one of *Hs*SUMO3 (amino acids 1–14) to incorporate its SCM. This chimeric SUMO protein was found to support the rapid build-up of HMW SUMO conjugates in the absence of *Aa*PIAS that was dependent upon the Lys acceptor residue (K11) within the SCM (Fig 2F). These data demonstrate that while *Aa*SUMO shares the highest degree of amino acid identity to *Hs*SUMO3, its biochemical activity as a ubiquitin-like protein is more functionally related to that of *Hs*SUMO1 due to the absence of a functional SCM.

**Fig 3:**
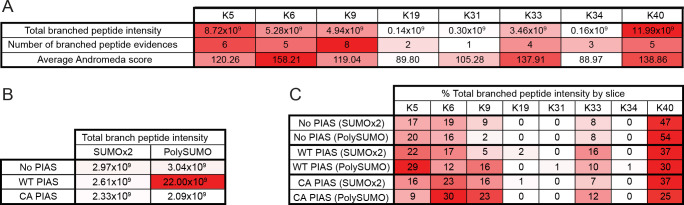
Internal lysine acceptor residues for *Aa*SUMO modification. (A) Summary of mass spectrometry data (S2 Fig) associated with branched SUMO peptides identified on SUMO Lys (K5, K6, K9, K19, K31, K33, K34, and K40) acceptor residues from *in vitro* reaction mixtures containing *Aa*SAE1/2, *Aa*Ubc9, and *Aa*SUMO. Data aggregated from all samples. MaxQuant Andromeda (peptide assignment score) values shown. (B) Total branched peptide intensity for all Lys acceptor residues from *in vitro* reaction mixtures (as in A) incubated in the presence or absence of WT or catalytically inactive (C371A, CA) *Aa*PIAS (as shown). (C) Slice-specific (dimeric SUMO, SUMOx2; HWM SUMO conjugates, polySUMO) peptide intensity data of Lys-specific acceptor residues. Data presented as a percentage of the total intensity of all branched peptides found in each gel slice. Representative spectra can be found in S7–S14 Figs. Data summary presented in S2 File. N = 3.

*Aa*SUMO contains eight Lys residues (Fig 1C) which could act as potential acceptor sites for SUMO modification in the presence of *Aa*PIAS. We next investigated which Lys residues within *Aa*SUMO were preferentially modified by *Aa*PIAS. Mass spectrometry analysis was conducted on SUMO-dimers and HMW poly-SUMO chains generated in the presence or absence of either WT or C371A *Aa*PIAS (S2 Fig). Spectral analysis demonstrated that *Aa*SUMO was preferentially conjugated to surface exposed Lys (K) residues K5, K6, K9, K40, and to a lesser extent, K33 (Figs 3A and S3 and S2 Files). Addition of *Aa*PIAS increased the proportion of detectible branched SUMO peptides, consistent with enhanced levels of poly-SUMO chain formation in a SP-RING dependent manner (Fig 2D and 2E) without strong preference for any specific solvent-exposed Lys residue (Figs 3B and 3C and S3). Collectively, these data demonstrate that *Aa*PIAS plays an important role in the SCM-independent formation of HMW *Aa*SUMO conjugates.

### *Aa*SUMO is differentially expressed in *Ae*. *aegypti* tissues

**Fig 4:**
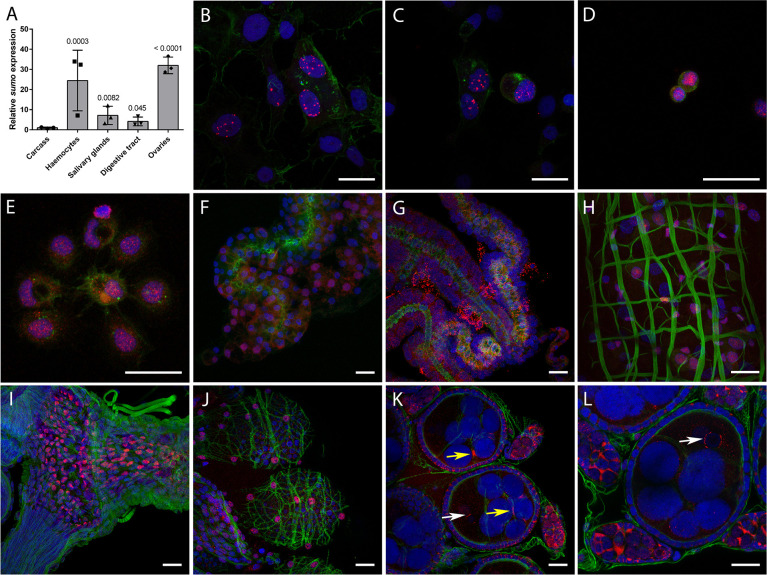
*Aa*SUMO is differentially expressed in *Ae*. *aegypti* tissues. (A) RNA was extracted from perfused haemocytes, dissected salivary glands, digestive tracts, ovaries, and carcasses (pools of 25 digestive tracts, ovaries or carcasses, 60 salivary glands, or pooled perfused haemocytes from 70 females per replicate experiment, N = 3 independent experiments). cDNA was synthesised and RT-qPCR was conducted to examine mRNA expression of *SUMO*. Data were analyzed as described by Taylor *et al*. (78). SUMO expression values were normalized to ribosomal S7 as a reference transcript, with a RQ geometric mean (geomean) of 1 for carcass. SD shown. Log2-normalized expression values were used for statistical analyses; one-way ANOVA (F_**4,10**_ = 17.99, p = 0.0001) followed by a Dunnett multiple comparison test relative to carcass (p values shown). (B to L) Representative confocal microscopy images showing the expression and sub-cellular localization of SUMO (red) in *Ae*. *aegypti* cells and tissues as detected by immunofluorescence assay. (B,C) AF5 cells; (D) perfused prohaemocytes; (E) perfused granulocytes; (F,G) salivary glands and surrounding fat body; (H) midgut; (I) oviduct; (J) ovarioles surrounded by the ovarian sheath; and (K,L) primary previtellogenic follicle and germarium inside ovarioles. Nuclei are stained by DAPI (blue) and F-actin is stained by Phalloidin 488 (green). Bar = 20 μm. Arrows indicate SUMO accumulation around the nucleus of the future oocyte (white) and at the perinuclear region of the nurse cells (yellow).

To determine if *Aa*SUMO was expressed in tissues relevant to arbovirus infection, we determined the relative expression levels of *SUMO* transcripts in multiple mosquito tissues from female mosquitoes; including salivary glands, digestive tract, ovaries, carcass (remaining abdominal tissue), and perfused haemocytes (mosquito immune cells). RNA was extracted and transcript expression levels analysed by RT-qPCR. *SUMO* transcripts were detected in all tissues and haemocytes, with significantly higher levels of *SUMO* expression observed in haemocytes and ovaries relative to the carcass (Figs 4A and S4).

We next examined the expression pattern of *Aa*SUMO protein in *Ae*. *aegypti*. Immunofluorescent confocal microscopy analysis was conducted using polyclonal antibodies raised to *Hs*SUMO2/3, which detect both unconjugated and conjugated *Aa*SUMO protein (Fig 2A and 2D). Microscopy assays were conducted on cultured *Ae*. *aegypti*-derived AF5 cells, perfused haemocytes, salivary glands, digestive tract, and ovaries from *Ae*. *aegypti* NBF females (Fig 4). Controls without primary antibody were also analysed under the same conditions to ensure signal specificity to *Aa*SUMO (S5 Fig). In AF5 cells, *Aa*SUMO was predominantly detected as nuclear punctate structures (Fig 4B and 4C). Consistent with our RT-qPCR results, *Aa*SUMO expression was detected in perfused haemocytes and in every *Ae*. *aegypti* tissue analysed. In haemocytes, *Aa*SUMO expression was restricted to the nucleus in prohaemocytes (Fig 4D), which have a high nuclear/cytoplasmic ratio [34], and differentiated granulocytes (Fig 4E), which are bigger cells with filopodia and a low nuclear/cytoplasmic ratio [34]. Very low levels of *Aa*SUMO expression were detected in the lobes of salivary glands (Fig 4F), with higher levels of *Aa*SUMO detected in surrounding fat body cells (Fig 4G). In the midgut, *Aa*SUMO was expressed in the nucleus of enterocytes (recognizable by their large nuclei) and in smaller enteroendocrine and/or stem cells (Fig 4H). In the ovaries, *Aa*SUMO was found to be highly expressed in the nuclei of the epithelial cells of the oviduct (Fig 4I). Similar staining was found in the ovarian sheath surrounding the ovarioles (Fig 4J). In the ovarioles, *Aa*SUMO was expressed within the primary previtellogenic follicle and in the germarium (Fig 4K and 4L). In the primary follicle, *Aa*SUMO was detected in the cytoplasm of the follicular cells surrounding the follicle. A strong circular signal of *Aa*SUMO protein was detected close to the nucleus of the oocyte (Fig 4K and 4L; white arrows) and at perinuclear regions of nurse cells (Fig 4K; yellow arrows). In the germarium, *Aa*SUMO was detected in the nucleus and cytoplasm of germline stem cells and follicle stem cells, in the cystoblasts as well as in the germline and follicular cells of the developing secondary follicle. Overall, our findings shows that *Aa*SUMO is differentially expressed in multiple tissues and cells known to support arbovirus replication, including the gut, ovaries, and haemocytes.

### The *Ae*. *aegypti* SUMOylation pathway has broad antiviral activity against arbovirus infection

**Fig 5:**
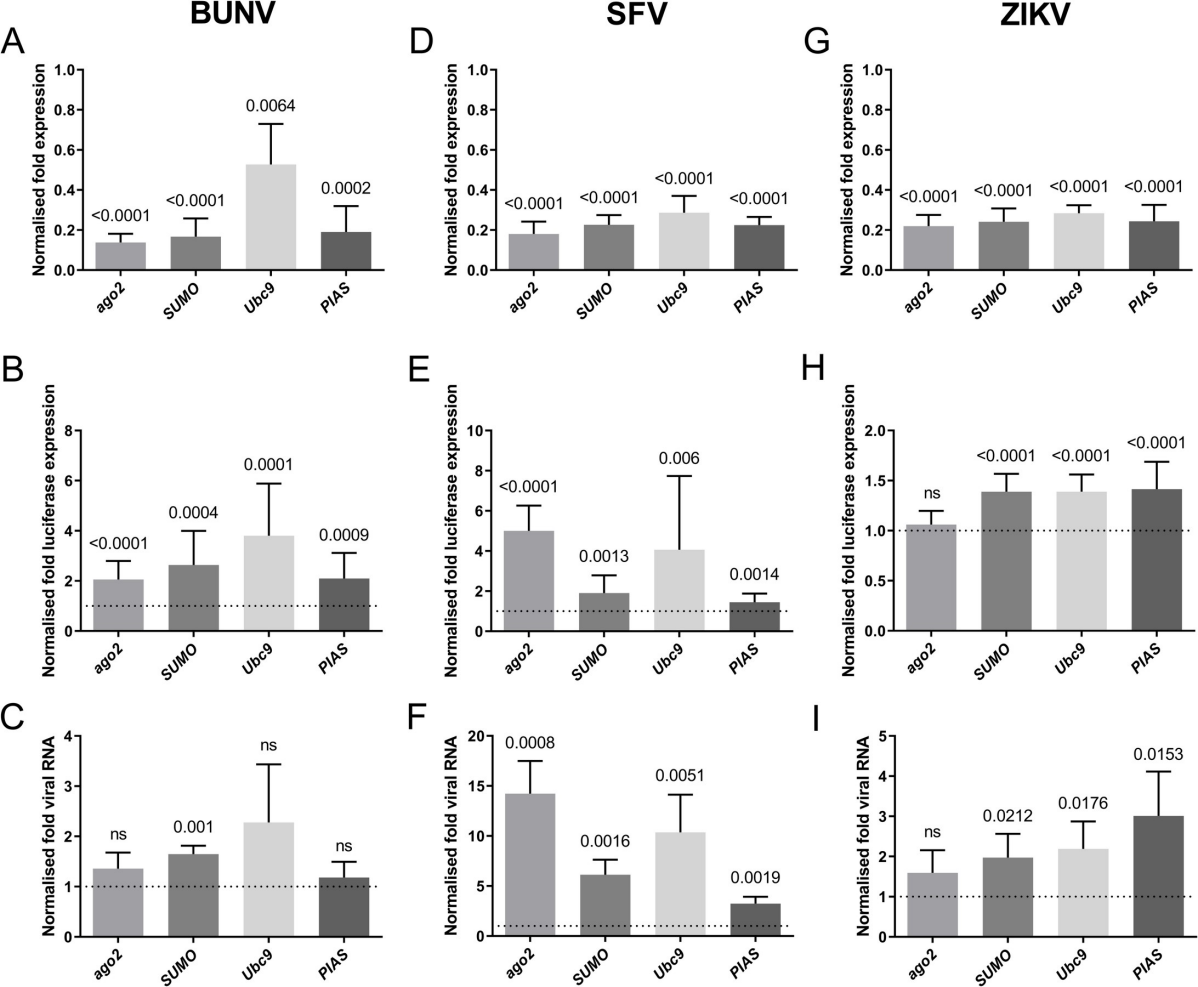
*Aa*SUMOylation effector proteins suppress arbovirus replication in *Ae*. *aegypti* cells. AF5 cells were treated with dsRNA targeting *SUMO*, *Ubc9*, *PIAS*, *LacZ* (non-target negative control), or a*go2* (positive control) for 72 hours prior to infection (MOI 0.05 PFU/cell) with BUNV, SFV, or ZIKV luciferase reporter viruses. Cells were harvested at 48 hours post-infection for luciferase and RT-qPCR analysis. (A,D,G) RT-qPCR analysis of mRNA levels of *ago2*, *SUMO*, *Ubc9*, or *PIAS* within infected AF5 cells. N = 5 independent biological replicates; values normalized to ribosomal *S7* and expressed relative to dsLacZ-treated control samples; RQ mean and SD plotted. (B, E, H) Luciferase readings from BUNV, SFV, or ZIKV infected samples. N = 15 independent biological replicates per condition; values expressed relative to dsLacZ-treated control samples (set to 1, dotted line); mean and SD plotted. (C, F, I) Viral RNA (vRNA) levels from BUNV, SFV, or ZIKV infected samples. N = 5 independent biological replicates; values expressed relative to dsLacZ-treated control samples (set to 1, dotted line); RQ mean and SD plotted. Statistical analysis, one sample (two-tailed) t test to a hypothetical mean of 1 (dsLacZ control), significant probability (P) values (≤ 0.05) shown; ns = not significant.

We next assessed the biological significance of the *Ae*. *aegypti* SUMOylation pathway during arbovirus infection. AF5 cells were treated with gene-specific (*AaSUMO*, *AaUbc9*, *AaPIAS*) or negative control (*lacZ*) dsRNA to transiently deplete SUMOylation effector proteins. As a positive control, AF5 cells were treated with a dsRNA targeting an effector of the exo-RNA interference (RNAi) pathway (*ago2*), which has known antiviral activity against SFV [35,36], BUNV [37], but not ZIKV [36,38,39]. Transfected cells were infected (MOI 0.05 PFU/cell) with luciferase reporter viruses from three independent arboviral families: BUNV (*Orthobunyavirus*, *Bunyaviridae*); SFV (*Alphavirus*, *Togaviridae*); or ZIKV (*Flavivirus*, *Flaviviridae*). These reporter viruses have been previously used to analyse the antiviral action of Ago2 and shown to be as genetically stable as their respective wild-type viruses (SFV [36,40–42]; BUNV [37]; ZIKV [43]). Transcript levels of *SUMO*, *Ubc9*, *PIAS*, and *ago2* were decreased from 50 to 80% relative to dsLacZ-treated control cells (Fig 5A, 5D and 5G), validating the efficiency of dsRNA depletion. As expected, depletion of Ago2 led to a significant increase in BUNV and SFV, but not ZIKV, replication relative to infected dsLacZ control cells (Fig 5B, 5E and 5H, respectively). Importantly, depletion of *Aa*SUMOylation pathway effectors (*Aa*SUMO, *Aa*Ubc9, and *Aa*PIAS) led to significantly higher levels of BUNV, SFV, and ZIKV replication independently of the effector protein targeted for depletion (Fig 5B, 5E and 5H, respectively). These data indicate that multiple component proteins of the *Aa*SUMOylation pathway contribute to the suppression of arbovirus replication; with BUNV and SFV being the most susceptible to *Aa*SUMOylation-mediated restriction. Significant increases in BUNV, SFV, and ZIKV intracellular RNA levels could also be observed in cells depleted for *Aa*SUMOylation proteins (Fig 5C, 5F and 5I, respectively); with SFV demonstrating the greatest increase in viral RNA (vRNA) levels (up to 10-fold relative to dsLacZ-treated control cells). It remains to be determined why depletion of *Aa*Ubc9 or *Aa*PIAS had no significant effect on the intracellular levels of BUNV RNA. However, this may reflect the relative level of effector protein depletion between replicate experiments (Fig 5A; Ubc9). In the majority of cases, disruption of the *Aa*SUMOylation pathway led to increases in both arbovirus replication (Fig 5B, 5E and 5H) and intracellular vRNA levels (Fig 5C, 5F and 5I). Collectively, these data indicate that the *Aa*SUMOylation pathway has a broad antiviral action against arboviruses from multiple families in *Ae*. *aegypti* cells.

## Discussion

The antiviral defences of mosquitoes are key determinants of vector competence, *i*.*e*. the ability of mosquitoes to get infected by an arbovirus following an infectious blood meal and subsequently transmit it to a new vertebrate host [44]. In mosquitoes, several pathways have been reported to play a role in the control of arbovirus infection. These include the RNAi and evolutionarily conserved innate immune pathways, such as the NF-κB signalling pathways (Toll and Imd) and the Janus kinase-signal transduction and activators of transcription (JAK/STAT) pathway [45,46]. Here, for the first time, we identify the *Ae*. *aegypti* SUMOylation pathway as being another evolutionarily conserved pathway that supresses arbovirus replication in mosquito cells.

To date, the majority of SUMO studies in arthropods has been conducted in *D*. *melanogaster*, which is known to possess a single SUMO gene (*smt3*; [47]). In contrast, two homologous SUMO genes have been previously identified in the *Ae*. *aegypti* genome [24,25]. Our analysis using the most recent annotated sequence [27] could only identify one SUMO gene (AAEL015064), a finding supported by our mass spectrometry analysis which identified unique peptides to all core effector proteins of the *Ae*. *aegypti* SUMOylation pathway (*Aa*SUMO, *Aa*SAE1/2, *Aa*Ubc9, and *Aa*PIAS). We demonstrate that *Aa*SUMO is highly related to *Hs*SUMO3, but lacks an N-terminal SCM restricting its ability to support poly-SUMO chain formation. Our analysis supports previous studies, which have suggested this motif to be lost in insects, but to be present in vertebrates and ticks [24,25]. Addition of *Aa*PIAS stimulated the accumulation of HMW *Aa*SUMO conjugates in an SP-RING dependent manner, demonstrating *Aa*PIAS to be a functionally conserved SUMO E3 ligase in mosquito cells. Notably, *Aa*PIAS promoted *Aa*SUMO conjugation onto multiple solvent exposed Lys residues, indicating that *Aa*PIAS may stimulate the formation of multiple or branched poly-SUMO chain types. This contrasts with poly-SUMO2/3 chain formation in vertebrate cells, which can occur independently of SUMO E3 ligase activity in a SCM K11-dependent manner [14]. This highlights an additional level of regulation required for the formation of poly-SUMO chains in *Ae*. *aegypti*, which are known to play many important biological roles in mammalian cells [16]. It remains to be determined if *Aa*PIAS, or other functionally conserved SUMO ligases, significantly contribute to poly-*Aa*SUMO chain formation *in vivo*. Our mass spectrometry and western blot analysis revealed that *Aa*SAE2 is SUMO modified *in vitro*, the levels of which are significantly enhanced in the presence of *Aa*PIAS in a SP-RING dependent manner. In *H*. *sapiens*, SUMO modification of SAE2 leads to altered protein sub-cellular localisation and reduced affinity for Ubc9 [32,33]. Thus, SAE2 auto-SUMOylation is likely to be a functionally conserved property of the SAE1/2 heterodimeric complex, although further studies are required to confirm the biological importance of this phenotype in *Ae*. *aegypti* cells.

The analysis of SUMO expression in NBF female *Ae*. *aegypti* reveals that SUMO is expressed in haemocytes (mosquito immune cells) and in various tissues, including the digestive tract, the ovaries, and the fat body. Our analysis demonstrated *SUMO* transcript to be ubiquitously expressed throughout *Ae*. *aegypti*, with the highest levels of expression in the ovaries and haemocytes. Of note, previous microarrays and RNA-seq studies have shown that *SUMO* expression levels in whole females do not change upon blood feeding and ZIKV infection [48,49]. Similar ubiquitous tissue expression of *SUMO* (*smt3*) in *D*. *melanogaster* has been found by RNA-seq, with an enrichment in ovaries (http://flyatlas.gla.ac.uk/FlyAtlas2/index.html). Although the expression of SUMO has yet to be reported in the haemocytes of adult flies, Ubc9 (lesswright, Lwr) is specifically required in larval haemocytes for haematopoiesis [50], suggesting that SUMO is expressed in *Drosophila* haemocytes. SUMO and Ubc9 are also ubiquitously expressed in the shrimp species *Fenneropenaeus chinensis* [51], with high levels of expression in haemocytes and ovaries. Thus, the tissue expression profile of SUMO in arthropods seems to be conserved across evolution. Indeed, Ubc9 is highly expressed in the reproductive organs and the blood cells in the fish *Cynoglossus semilaevis* [52] and in immune cells, endocrine tissues, reproductive tissues, and adipose tissues in *H*. *sapiens* (Human Protein Atlas, https://www.proteinatlas.org/) [53,54].

In many of the *Ae*. *aegypti* tissues and cells studied here, SUMO is predominantly expressed in punctate structures in the nuclei. This pattern of expression in mammalian cells is due to the extensive SUMO modification of Promyelocytic leukaemia nuclear bodies (PML-NBs), a process required for PML-NB formation and associated protein-protein interactions [55]. PML-NBs are involved in many cellular processes, including the cell cycle, genome stability, and antiviral defence, through the SUMO-dependent sequestration, modification, and degradation of associated host factors [56]. The PML gene is not evolutionarily conserved among eukaryotes, being absent in lower eukaryotes such as insects [57]. However, as many other RING containing proteins (including other TRIM family members like PML; TRIM19) can form nuclear structures, including in insects [58], it is possible that SUMO modification of other RING proteins is involved in the formation of nuclear complexes in mosquito cells with similar regulatory functions.

SUMO modification is known to regulate the biological function of hundreds of cellular proteins [11]. Previous studies have clearly established a role of SUMOylation, both positive and negative, in the replication of DNA and RNA viruses in mammalian cells [17–19]. The SUMOylation pathway is also known to influence arbovirus replication in *H*. *sapiens* cells, although its pro- or antiviral action is still a matter of debate and warrants additional study [20–23]. We have now shown that the SUMOylation pathway influences replication of a range of arboviruses in *Ae*. *aegypti* cells. Indeed, depletion of components of the SUMOylation pathway led to a consistent increase in viral replication and intracellular RNA levels of BUNV, SFV, and ZIKV; albeit to varying extents. Such differences may reflect virus-specific interfaces with the *Aa*SUMOylation pathway that requires further study. Importantly, depletion of SUMO effector proteins led to an increase in viral replication of BUNV and SFV equivalent to that of Ago2 depletion, a key mediator of the *Ae*. *aegypti* antiviral RNAi pathway [46]. Thus, we identify the *Aa*SUMOylation pathway to contribute to the antiviral immune response to arbovirus infection in mosquito cells. However, we cannot discount a positive (pro-viral) role for *Aa*SUMOylation, as there may well be virus-specific requirements for host SUMOylation in certain mosquito cell types or tissues. Notably, *Aa*PIAS depletion had an overall weaker effect on SFV replication in mosquito cells relative to that of Ubc9 depletion. Such differences may be linked to virus-specific requirements for component proteins of the *Aa*SUMOylation pathway to restrict arbovirus replication. Previous studies suggest that members of the *H*. *sapiens* PIAS family of E3 ligases, which are primarily known for their role as negative regulators of innate immunity, also possess intrinsic immune functions to suppress viral replication in mammalian cells [59–61]. In *Ae*. *aegypti*, transient depletion of *Aa*PIAS decreased DENV and ZIKV infection intensity in the midgut [62,63], consistent with PIAS proteins being negative regulators of the JAK/STAT pathway. By way of contrast, activation of the JAK/STAT pathway in the mosquito fat body decreased DENV infection while it had no effect on ZIKV and CHIKV [64]. These data suggest virus- or tissue-specific roles for the JAK/STAT pathway in the immune regulation of arbovirus infection in mosquito cells, which may account for why we did not observe an overall net decrease in arbovirus replication following *Aa*PIAS depletion in our study.

Collectively, our results demonstrate that the sequence, function, and tissue distribution of the *Ae*. *aegypti* SUMOylation pathway effector proteins are highly conserved. We have shown the SUMO pathway to suppress arbovirus replication from three independent viral families. Thus, we identify SUMOylation as an additional conserved pathway involved in antiviral defences in cells derived from *Ae*. *aegypti*. Our data helps to fill many long-standing gaps in the literature about the conservation and biochemical properties of the SUMOylation pathway in this transmitting vector species, and the overall net functional effect of *Ae*. *aegypti* SUMO modification during arbovirus replication. Areas for future research include determining in which tissues and cell types, where arboviruses can replicate (*e*.*g*. gut, salivary glands, and haemocytes; [65–67]), and how, mosquito SUMOylation controls arbovirus replication. Further analyses using tissue-specific genetic tools and vector-specific antibodies will be required to assess the precise location and mechanisms of restriction in *Ae*. *aegypti*.

SUMOylation can influence the function of a wide variety of proteins and cellular processes. Nevertheless, some hypotheses explaining the antiviral action of SUMOylation in mosquito cells can be proposed. In *D*. *melanogaster*, SUMOylation occurs on diverse immune proteins that can modulate innate immune function, including JAK/STAT and Imd patways [26,68,69]. In arbovirus (BUNV, SFV, and ZIKV) infected mosquito cells silenced for Ubc9, we failed to observe a significant decrease of *vir-1* (JAK-STAT effector, [70]) or *cecropin D* (Imd effector, [71] expression (S6 Fig), which suggests that *Aa*SUMOylation does not directly influence the transcription of these effector proteins. However, we cannot exclude that *Aa*SUMOylation could enhance the expression, stability, or function of other innate immune regulators to mediate an antiviral effect in mosquito cells. Alternatively, arboviral proteins may be targeted directly for SUMOylation to modulate their function or stability, as observed for the DENV NS5 protein in mammalian cells [23]. Further studies are warranted to determine the precise mechanism(s) associated with the antiviral role(s) of host SUMOylation in *Ae*. *aegypti* cells that suppress arbovirus replication.

## Materials and methods

### Cell culture

*Ae*. *aegypti* cell lines A20 and AF5 [40,72,73] were maintained in Thermo Fisher Scientific Nunclon Delta surface (25 cm^2^) tissue culture flasks with Leibovitz’s L-15 media (Gibco) supplemented with 10% (w/v) Tryptose phosphate broth (TPB; Gibco) and 10% (w/v) Foetal Bovine Serum (FBS; Gibco). BHK-21 cells (obtained from Roger D. Everett [MRC-UoG CVR]) were cultured in GMEM (Gibco), supplemented with 10% TPB and 10% FBS, Vero-E6 cells (obtained from Claire Donald [MRC-UoG CVR]) were cultured in DMEM (Gibco) supplemented with 10% FBS. A549-Npro cells (obtained from Claire Donald [MRC-UoG CVR]) were also cultured in DMEM (Gibco), supplemented with 10% FBS and 0.5 μg/ml puromycin. A549-Npro, BHK-21 and Vero-E6 cells were grown at 37°C with 5% CO_2_, A20 and AF5 cells were cultured at 28°C.

### Virus stocks

Semliki Forest virus (SFV) with a firefly luciferase reporter (SFV4-*FFluc* described in [74]) was a kind gift from Dr. Esther Schnettler (MRC-UoG CVR) and grown and titrated in BHK-21 cells as previously described [42]. Stocks of BUNV encoding a NanoLuciferase reporter protein (BUNV-NLuc described in [37]) was a kind gift from Dr. Xiaohong Shi (MRC-UoG CVR) and grown and titrated in BHK-21 cells as previously described [75]. ZIKV encoding a NanoLuciferase protein (ZIKV-NLuc described in [43]) was produced and kindly gifted by Jamie Royle (MRC-UoG CVR) grown and titrated in Vero-E6 and A549-Npro cells, respectively as previously described [43,76].

### Plasmids

*Aa*SAE1/2 were amplified from A20 cDNA using gene-specific primers (S1 Table). CDS were cloned into a pACYCDuet-1 dual expression vector (Sigma-Aldrich; Merck KGaA) using EcoRI/NotI (SAE2) and NdeI/XhoI (SAE1) unique sites. A Strep.II tag (5’-TGGAGCCACCCGCAGTTCGAAAAG-3’) was synthesised (Dundee Cell Products) and inserted to replace 6xHis tag sequences between NcoI-EcoRI in frame with *Aa*SAE2. WT SUMO chimaera (amino acids 1–14 of *Hs*SUMO3 and 14–91 of *Aa*SUMO) and mutant SUMO chimaera (K11R) CDS were synthesised (GENEWIZ), amplified with the primers ‘pET28-AaSUMO R’ and ‘pET28-SUMO chimaera F’ (S1 Table), and inserted into pET28a (Novagen) using NdeI/XhoI restriction sites. A CDS for *Aa*SUMO (cloned from A20 cells; a kind gift from Dr. Sue Jacobs, Pirbright Institute) was cloned into pET28a using primers ‘pET28-*Aa*SUMO F’ and ‘pET28-*Aa*SUMO R’ (S1 Table) using NdeI/XhoI restriction sites. WT and mutant (C371A) *Aa*PIAS CDS were synthesised (GENEWIZ), amplified by PCR with the primers ‘*Aa*PIAS F’ and ‘*Aa*PIAS R’ (S1 Table), and cloned into a pET45b (Sigma-Aldrich; Merck KGaA) using AgeI/NotI restriction sites. *Aa*Ubc9 was cloned from A20 cDNA using primers ‘pET45-*Aa*Ubc9 F’ and ‘pET45-*Aa*Ubc9 R’ (S1 Table) and cloned into pET45b using AgeI/XhoI restriction sites. Bacterial expression vectors for *Hs*SAE1/2, *Hs*SUMO1, *Hs*SUMO2, *Hs*SUMO3, and *Hs*Ubc9 are described in [14]. All restriction enzymes were purchased from New England Biolabs. All final constructs were confirmed by Sanger sequencing (Source Bioscience).

### Protein expression and purification

BL21 (DE3) cells were transformed with a bacterial expression plasmid containing the CDS of interest under the appropriate antibiotic selection. Single colonies were grown in 10 ml of Luria Bertani Broth (LB) O/N at 37°C in an orbital shaker (200 RPM). 5 ml was added to 200 ml of LB and allowed to grow for a further 2 hours at 37°C, 200 RPM. Protein expression was induced with 0.1 mM isopropyl-thio-β-D-galactoside. Induction occurred in an orbital shaker at 30°C, 200 RPM for 4–5 hours. Bacteria were pelleted by centrifugation at 3500 x *g* for 10 min at 4°C. Unless otherwise stated, the bacterial pellets were resuspended in 25 ml 6xHis Purification Buffer (50 mM Tris (pH7.5), 250 mM NaCl, 10 mM β-mercaptoethanol, 5% glycerol, 0.1% Triton-X-100, 2.5 mM MgCl_2_, 30 mM imidazole and a protease inhibitor cocktail (Roche, pH 7.5). Bacteria were lysed by probe-sonication (45 pulses, 30 Amps) and centrifuged (13,000 x *g* for 10 min). Bacteria containing pACYC-Strep.II-*Aa*SAE2/1 were resuspended in 2 ml Strep Purification Buffer (50 mM Tris pH 7.0, 50 mM NaCl, 1 mM MgCl_2_, and a protease inhibitor cocktail (Roche, pH 7.5). Samples were then lysed by digital sonicator (7 repeats at 50% intensity for 30 sec, at 4°C) and centrifuged (14,000 x *g* for 10 min). Supernatants containing recombinant proteins were filtered through a 0.45 μm filter, bound to 250 μl (w/v) of equilibrated Ni-NTA (Qiagen) or Strep-tactin Superflow agarose beads (Novagen) in the corresponding purification buffers. Samples were tumbled end-over-end for 90 min at room temperature (RT), beads sedimented centrifugation (1500 x *g* for 5 min), washed twice in 10 ml of purification buffer and three times in 1 ml of purification buffer. 6xHis tag proteins were eluted in 750 μl of 6xHis Purification Buffer containing 350 mM (final concentration) imidazole at pH 7.5 prior to dialysis in Dialysis Buffer (50 mM Tris (pH 7.5), 150 mM NaCl, 5% glycerol, 2 mM MgCl_2_ and 5 mM β-mercaptoethanol, 0.1% Triton-X-100) for 4 hours. Strep.II-*Aa*SAE2/1 was eluted in 600 μl Strep Purification Buffer with 2.5 mM D-desthiobiotin (pH 8.5) prior to dialysis in Dialysis Buffer for 4 hours. Proteins were visualized by SDS-PAGE (4–12% gradient PAGE; Invitrogen) alongside purified commercial BSA standards (BioRad) by Coomassie brilliant blue staining. Protein concentration was determined on the Li-Cor Odyssey Imaging System.

### Biochemical assays

Master mixes of *Ae*. *aegypti* or *H*. *Sapiens* recombinant SUMO pathway component proteins were aliquoted into individual tubes containing SUMO, Ubc9, and SAE1/2 (50 ng each/reaction), and if required, WT or mutant (C371A) PIAS (10 ng/reaction). The reaction was initiated by addition of SAE1/2 and incubated at 37 or 28°C for the desired length of time. The reaction was terminated by the addition of 1.5xBM (Laemmli buffer supplemented with 2.6 M Urea and 50 mM Dithiothretol). This sample mixture was boiled at 95°C for 10 min prior to SDS-PAGE and western blotting. Biochemical assays for Mass spectrometry analysis were performed with 5 μg SUMO, 500 ng Ubc9, 500 ng SAE1/2, and 50 ng WT or C371A PIAS, and incubated at 28°C for 1 hour. Reactions were terminated by the addition of NuPAGE Sample Reducing Agent (Invitrogen).

### Western blots

Samples were resolved on a NuPAGE-Novex 4–12% Bis-Tris Gradient gel (Invitrogen) with 1X NuPage MES SDS running buffer (Invitrogen). PAGE gels were transferred onto an Amersham protran 0.2 μm nitrocellulose blotting membrane (GE Healthcare) in transfer buffer (1x NuPage transfer buffer and 10% Methanol) at 30 V for 60 min. The membranes were blocked in blocking buffer (5% FBS in PBS) for 1 hour. The mouse anti-SUMO2/3 primary antibody (Cat. No. Ab81371; Abcam) diluted 1:1,000 in blocking buffer was incubated on the blotting membrane for 1 hour or O/N at 4°C. The membrane was washed 3 times for 5 min in PBST (0.1% Tween-20). A DyLight800 fluorescent goat anti-mouse secondary antibody (SA535521; Thermo Fisher Scientific) diluted 1:10,000 was incubated with the membrane for 1 hour. All steps were conducted at room temperature unless stated otherwise. Membranes were imaged and quantified using a LiCor Odyssey Imaging System.

### dsRNA production

Total RNA was extracted from *Ae*.*aegypti*-derived A20 cells using an RNAeasy Plus Kit (Qiagen), following manufacturer’s instructions. cDNA was synthesised with TaqMan Reverse Transcription Reagents kit (Life Technologies) with Random Hexamer primers according to the manufacturer’s instructions. To synthesise dsRNA, cDNA was amplified with gene-specific primers (S1 Table) incorporating the T7 RNA polymerase promoter sequence at the 5’ end. PCR was conducted using Phusion polymerase (New England Biolabs), PCR products were excised from the gel using QIAquick Gel Extraction Kit (Qiagen), and sequenced to confirm identity by Sanger sequencing (Source Bioscience). dsRNA were produced using the MEGAscript RNAi kit (Ambion) according to the manufacturer’s instructions. The dsRNA were quantified using a Nanodrop spectrophotometer.

### dsRNA transfection and infection

AF5 cells (2.5x10^5^ / well) were seeded in 24-well plates and left in the incubator overnight (O/N) at 28°C prior to the transfection of 300 ng dsRNA with 2 μl DharmaFECT2 (Dharmacon) per well, following manufacturer’s instructions. Cells were incubated for 72 hours prior to infection (MOI of 0.05 PFU per cell). After 48 hours, cells were harvested for RT-qPCR (viral load and depletion efficiency) or luciferase analysis.

### Luciferase assay

Luciferase expression was determined using the Luciferase Assay System Kit (Promega) for SFV-*Ffluc* and the Nano-Glo Luciferase Assay System Kit (Promega) for BUNV-NLuc and ZIKV-NLuc. Luciferase activities were determined on a GloMax 20/20 Luminometer following the manufacturer’s instructions. A minimum of 5 independent biological repeats were conducted in triplicate for each experimental condition.

### Mosquito rearing

*Ae*. *aegypti* mosquitoes (Liverpool strain, gift from Pr. Eileen Devaney, University of Glasgow) were reared at 28°C and 80% humidity with a 12:12 light photoperiod. Larvae were reared in water with some dry pieces of cat food pieces from larvae hatching to pupal stage. Emerging adult mosquitoes were transferred in cages with unlimited access to 10% w/v sucrose solution. For rearing, female mosquitoes were fed on rabbit blood supplemented with heparin (Orygen Antibodies Ltd) using a Hemotek (Hemotek Ltd., UK) at 37°C.

### Dissection of mosquito tissues and perfusion of haemocytes

Tissues were dissected from 5 day-old females in RNase free 0.05% PBS-Tween 20 (PBST) (v/v). For RNA extraction, tissues were stored at -80°C. For immunostainings, tissues were stored in PBS-T 0.05% on ice before fixation. Haemocytes were collected by perfusion. The last segment of the abdomen was cut and mosquitoes were then injected in the thorax with 0.01% PBS-T. For RT-qPCR experiments, PBS-T diluted haemocytes were collected in tubes on ice, further centrifuge at 2,000 RPM for 15 min at 4°C. The supernatant was removed before the addition of TRIzol reagent (Thermo Fisher Scientific). For immunostainings, haemocytes were perfused on slides (ibidi) which were incubated for 30 min at 28°C before removal of PBS-T for fixation and staining.

### RNA extraction and reverse transcription (RT)-quantitative PCR

Total RNA was isolated from cells using the RNAeasy Plus Kit (Qiagen), following manufacturer’s instructions. cDNA was synthesised with TaqMan Reverse Transcription Reagents kit (Life Technologies) with Random Hexamer primers, as per manufacturer’s instructions. Samples were analysed in triplicate using SYBR Green Master mix (Applied Biosystems) with gene-specific primers (S1 Table). Quantitative PCR (qPCR) was conducted on an Applied Biosystems 7500 Fast-Real-Time PCR system using MicroAmp plates and Optical Adhesive covers (Applied Biosystems). Relative mRNA expression levels were determined using the 2^−ΔΔCt^ (cycle threshold) method [77], normalised to the *S7* ribosomal gene and presented as relative values to that of the dsLacZ control. Data presented for the knockdown efficiency and the viral loads is the mean of 5 independent biological repeats. RQ means and standard deviations are presented.

Dissected mosquito tissues (pools of 25 digestive tracts, ovaries or carcasses, pools of 60 salivary glands, and pools of perfused haemocytes from 70 females per independent replicate) were homogenised in TRIzol (Thermo Fisher Scientific) with glass beads using the Precellys 24 homogeniser (Bertin Instruments). RNA from tissues and haemocytes were further extracted using the Trizol method according to the manufacturer’s instructions except that 1-Bromo-3-chloropropane (Sigma) was used instead of chloroform and DNase (TURBO DNase, Ambion) treatment was performed. Reverse-transcriptions (RT) and RT negative controls were performed using the MMLV retro-transcriptase (Promega) from 25 ng/μl of RNA according to the manufacturer’s instructions. cDNAs were aliquoted and stored at -20°C until qPCR. qPCR was performed using Fast SYBR Green master mix (Applied Biosystems) following the manufacturer’s instructions using the 7500 Fast machine (Applied Biosystems). Primers used are listed in S1 Table. Results were analysed with the 7500 Software v2.0.6. Data were analyzed as described by Taylor *et al*. [78] to obtain normalized expression values, relative to ribosomal S7 transcript expression, with a geometric mean (geomean) of 1 for the carcass condition. Log2-normalized expression values were used for statistical analyses.

### Immunofluorescence assays

Salivary glands and ovaries (n≥10 of each per replicate) were fixed at room temperature for 20 min in 4% (w/v) paraformaldehyde (PFA) diluted in PBS-T 0.05%. AF5 cells (3x10^4^ cells/well, incubated O/N at 28°C on ibidi slides), perfused haemocytes (from a pool of 5 females per replicate) and guts (n≥10 per replicate) were fixed the same way with the exception that the PFA was diluted using PBS. Fixed haemocytes/tissues (except ovaries) were washed (three times, 15 min each at 4°C) in PBS-T 0.05%, before being blocked for a minimum of 30 min in blocking solution (0.25% PBS-T 5% FBS (v/v), 5% BSA (w/v), 0.25% Triton X-100 (v/v) at 4°C. Unlike other tissues, ovaries were dilacerated and washed in 1% PBS-T before being blocked in blocking solution containing 0.5% Tween and 0.5% Triton. AF5 cells, haemocytes, and tissues were incubated at 4°C overnight with a mouse anti-SUMO2/3 antibody (Cat. No. Ab81371; Abcam) diluted 1:1000 in blocking solution. For each experiment, a negative control without primary antibody was performed. Samples were washed (five times, 15 min each at 4°C) in 0.05% PBS-T (0.5% PBS-T for ovaries) and incubated with an Alexa Fluor 568 goat anti-mouse IgG (H+L) diluted 1:1,000, DAPI 1X (405 nm) and Phalloidin 1X (488 nm) in blocking solution for 2 hours at RT. Four washes in 0.05% PBS-T (0.5% PBS-T for ovaries) were done and tissues were mounted between a slide and coverslip (24 mm x 24 mm) with an imaging spacer (1 well, diam. x thickness 13 mm x 0.12 mm Grace Bio-Labs SecureSeal imaging spacer, Sigma-Aldrich) using ibidi Mounting Medium (ibidi). Mounting media was used to replace the PBS-T in haemocytes. Images were acquired on a Zeiss LSM 710 inverted confocal microscope, equipped with a 40X or 63X oil-immersion objective and processed with Fiji/ImageJ and Adobe Photoshop. Two independent replicates were performed.

### Shotgun proteomic analysis to identify expressed SUMO pathway proteins

AF5 cell extracts were harvested in 1.5XBM and proteins fractionated by 4–12% Bis-tris SDS-PAGE gel running in MES buffer. Each lane was excised into 12 slices and tryptic peptides extracted as described previously [79]. All 132 peptide samples were analyzed by LC-MS/MS on a Q Exactive mass spectrometer (Thermo Scientific) coupled to an EASY-nLC 1000 liquid chromatography system via an EASY-Spray ion source (Thermo Scientific) running a 75 μm x 500 mm EASY-Spray column at 45°C. Peptides were fractionated over a 90 minute gradient and data were acquired in the data-dependent mode. Full scan spectra (m/z 300–1800) were acquired with resolution R = 70,000 at m/z 200 (after accumulation to a target value of 1,000,000 with maximum injection time of 20 ms). The 10 most intense ions were fragmented by HCD and measured with a resolution of R = 17,500 at m/z 200 (target value of 500,000, maximum injection time of 60 ms) and intensity threshold of 2.1x10^4^. Peptide match was set to ‘preferred’ and a 40 second dynamic exclusion list was applied. The resultant 132 raw MS data files were processed using MaxQuant (v 1.6.1.0) with the built-in Andromeda peptide search engine [80]. The *Ae*. *aegypti* uniprot protome (downloaded August 2017) was searched. Enzyme specificity was set to trypsin-P. Cysteine carbamidomethylation was selected as a fixed modification with methionine oxidation and protein N-terminal acetylation as variable modifications. Initial maximum allowed mass deviation was set to 20 parts per million (ppm) for peptide masses and 0.5 Da for MS/MS peaks. The minimum peptide length was set to 7 amino acids and maximum size 4600 Da. A maximum of two missed cleavages were considered. A false discovery rate (FDR) of 1% was required at both the protein and peptide levels. The ‘match between runs’ option was applied with a time window of two minutes matching between samples from the same gel slice position across different lanes, or a slice above or below. 5462 protein groups were identified after removal of decoy proteins, putative contaminants and those only identified by modified peptide. Peptide level data for SUMO, Ubc9, SAE1, SAE2 and PIAS proteins are shown in S1 File.

### Shotgun proteomic analysis of *in vitro* SUMO conjugation reactions to identify sites of polymerisation

Three *in vitro* SUMO conjugation reactions were prepared in triplicate: without any E3, with WT PIAS, or with C371A mutant PIAS (see above). Proteins were fractionated by 10% Bis-tris SDS-PAGE gel running in MOPS buffer. Each lane was excised into upper and lower slices to gain information on SUMO-SUMO dimers, and high molecular weight polymers. Tryptic peptides were extracted as described above. Half of each tryptic peptide sample was further subjected to in solution digestion with GluC to shorten the C-terminal SUMO adduct attached to substrate peptides, hence increasing likelihood of detection of as many SUMO conjugation sites as possible. Two mass spectrometry runs were performed for tryptic peptides and one for the doubly digested Trypsin-GluC peptides. Instrument setting were essentially as described above except for the second trypic peptide run and the only Try-GluC run for which a top1 method was applied with a 1000ms fill time. These settings improve MS/MS spectra for large low abundance peptides. To automatically detect branched peptides a concatenated database approach was taken, similar to previously described [81]. Briefly, a sequence file in fasta format was generated whereby the C-terminus of SUMO was concatenated to all conceivable peptides yielded by protease digestion of SUMO, Ubc9, SAE1 and SAE2. This concatenated sequence database contains individual ‘proteins’ that mimic the SUMO-substrate branched peptides allowing MaxQuant to automatically search for them in the MS data. MaxQuant was set up as described above except the concatenated peptide fasta file was used along with a file containing all proteins present in the *in vitro* reactions, GluC and Try/P (6 missed cleavages) or just Try/P alone (4 missed cleavages) were defined as proteases, and max peptide mass was set to 10000. Representative spectra can be found in S7–S14 Figs and data summary can be found in S2 File.

### Bioinformatics and statistical analysis

Amino acid alignment was conducted using the online software T-COFFEE (http://tcoffee.crg.cat/) [82]. These results were then plotted and exported with BOXSHADE (https://embnet.vital-it.ch/software/BOX_form.html). Models of *Aa*SUMO were conducted by obtaining the annotated amino acid sequence from UniProt, and predicting the tertiary structure with Phyre2 and plotting the structure in Chimera 1.10.1 [83,84]. Statistical analysis and graphs were produced using GraphPad Prism 7.02. Statistical tests are indicated in figure legends.

## Supporting information

S1 Fig Recombinant SUMOylation pathway proteins. 6xHis- or Strep.II-tagged proteins of the (A) *H*. *sapiens* and (B) *Ae*. *aegypti* SUMOylation pathways were expressed in bacteria and purified through Nickle- or Biotin-affinity chromatography. Samples were resolved by SDS-PAGE and Coomassie stained. A BSA gradient is included to assess concentration. Molecular weights indicated.
(JPG)

S2 Fig Schematic of the mass spectrometry protocol. Coomassie gel showing *in vitro* conjugation assay products from reactions either lacking *Aa*PIAS (-), or including wild-type (WT) or inactive C371A mutant variants (CA). Schematic explaining sample processing and mass spectrometry analysis using a branched peptide database is shown.
(JPG)

S3 Fig Predicted location of acceptor lysine residues with *Aa*SUMO. SUMO internal lysine residues identified as being prominent acceptors of SUMO modification in Fig 3 were plotted on the predicted structure of *Aa*SUMO (blue). Green indicates poor acceptors of SUMO modification (total branched peptide intensity <3x10^9^), while magenta indicates the lysine residues which are predominantly modified (total branched peptide intensity >3x10^9^). K19, K31, and K34 are shown in green, while K5, K6, K9, K33, and K40 are in magenta. (A) Front view. (B) Top view. (C, D) side view rotated ± 90° from A.
(JPG)

S4 Fig *AaSUMO* is differentially expressed in *Ae*. *aegypti* tissues. Data presented on Fig 4A were subjected to an ANOVA test (F_4,10_ = 17.99, p = 0.0001) followed by a Tukey’s post hoc multiple comparison test. Letters above bars indicate post hoc significance. Groups with the same letter are not significantly different. Table shows p values for each group comparison.
(JPG)

S5 Fig No primary antibody control IFA in haemocytes and tissues of females *Aedes aegypti*. Immunofluorescence assay without primary antibody on perfused haemocytes (A), salivary glands (B), midgut (C) and ovaries (D). The signal was revealed by an Alexa Fluor 568 goat anti-mouse IgG (H+L) diluted 1:1000 (red). Nuclei are stained by DAPI (blue) and F-actin is stained by Phalloidin 488 (green). The images were acquired on a Zeiss LSM 710 inverted confocal microscope with 40X or 63X oil-immersion objective and using the same parameters as those used for samples incubated with anti-SUMO primary antibody (Fig 5). Scale bar is 20 μm.
(JPG)

S6 Fig Expression of *vir1* and *cecropin D* in *Ae*. *aegypti* infected cells depleted for Ubc9. Expression of *vir1* and *cecropin D* (*cecD*) was analysed in dsUbc9- and dsLacZ-treated cells (as described in Fig 5) by RT-qPCR. N = 5 independent biological replicates. Values normalized to ribosomal *S7* and expressed relative to dsLacZ-treated control samples set to 1; RQ mean and SD plotted. Statistical analysis, one sample (two-tailed) t test to a hypothetical mean of 1 (dsLacZ control), significant probability (P) values (≤ 0.05) shown. (A, B, C) Expression of *vir1* in BUNV-, SFV- and ZIKV-infected cells, respectively. (D, E, F) Expression of *cecropin D* in BUNV-, SFV- and ZIKV-infected cells, respectively.
(JPG)

S7 Fig Evidence for *Aa*SUMO polymerisation at lysine 5. (A, B) Best spectra for two different peptides providing evidence for polymerisation of *Aa*SUMO via lysine 5. See S2 File for details. Note, due to the use of a concatenated database of N-terminal fusions of the SUMO C-terminus to potential substrate peptides, many fragments are not annotated. These are mostly b series ions from the substrate peptide up to the modified lysine.
(JPG)

S8 Fig Evidence for *Aa*SUMO polymerisation at lysine 6. (A, B) Best spectra for two different peptides providing evidence for polymerisation of *Aa*SUMO via lysine 6. See S2 File for details. Note, due to the use of a concatenated database of N-terminal fusions of the SUMO C-terminus to potential substrate peptides, many fragments are not annotated. These are mostly b series ions from the substrate peptide up to the modified lysine.
(JPG)

S9 Fig Evidence for *Aa*SUMO polymerisation at lysine 9. (A, B) Best spectra for two different peptides providing evidence for polymerisation of *Aa*SUMO via lysine 9. See S2 File for details. Note, due to the use of a concatenated database of N-terminal fusions of the SUMO C-terminus to potential substrate peptides, many fragments are not annotated. These are mostly b series ions from the substrate peptide up to the modified lysine.
(JPG)

S10 Fig Evidence for *Aa*SUMO polymerisation at lysine 19. (A, B) Best spectra for two different peptides providing evidence for polymerisation of *Aa*SUMO via lysine 19. See S2 File for details. Note, due to the use of a concatenated database of N-terminal fusions of the SUMO C-terminus to potential substrate peptides, many fragments are not annotated. These are mostly b series ions from the substrate peptide up to the modified lysine.
(JPG)

S11 Fig Evidence for *Aa*SUMO polymerisation at lysine 31. Best spectrum for the only peptide providing evidence for polymerisation of *Aa*SUMO via lysine 31. See S2 File for details. Note, due to the use of a concatenated database of N-terminal fusions of the SUMO C-terminus to potential substrate peptides, many fragments are not annotated. These are mostly b series ions from the substrate peptide up to the modified lysine.
(JPG)

S12 Fig Evidence for *Aa*SUMO polymerisation at lysine 33. (A, B) Best spectra for two different peptides providing evidence for polymerisation of *Aa*SUMO via lysine 33. See S2 File for details. Note, due to the use of a concatenated database of N-terminal fusions of the SUMO C-terminus to potential substrate peptides, many fragments are not annotated. These are mostly b series ions from the substrate peptide up to the modified lysine.
(JPG)

S13 Fig Evidence for *Aa*SUMO polymerisation at lysine 34. (A, B) Best spectra for two different peptides providing evidence for polymerisation of *Aa*SUMO via lysine 34. See S2 File for details. Note, due to the use of a concatenated database of N-terminal fusions of the SUMO C-terminus to potential substrate peptides, many fragments are not annotated. These are mostly b series ions from the substrate peptide up to the modified lysine.
(JPG)

S14 Fig Evidence for *Aa*SUMO polymerisation at lysine 40. (A, B) Best spectra for two different peptides providing evidence for polymerisation of *Aa*SUMO via lysine 40. See S2 File for details. Note, due to the use of a concatenated database of N-terminal fusions of the SUMO C-terminus to potential substrate peptides, many fragments are not annotated. These are mostly b series ions from the substrate peptide up to the modified lysine.
(JPG)

S1 Table Primer sequences. (DOCX)

S1 File Peptide level data for SUMO, Ubc9, SAE1, SAE2 and PIAS proteins in *Ae*. *aegypti* AF5 cells. (XLSX)

S2 File Data summary of shotgun proteomic analysis of *in vitro* SUMO conjugation reactions to identify sites of polymerisation. (XLSX)

## References

[1] Weaver SC, Charlier C, Vasilakis N, Lecuit M. Zika, Chikungunya, and Other Emerging Vector-Borne Viral Diseases. Annu Rev Med. 2018;69:395–408. 10.1146/annurev-med-050715-105122 28846489 PMC6343128

[2] Messina JP, Brady OJ, Golding N, Kraemer MUG, Wint GRW, Ray SE, et al The current and future global distribution and population at risk of dengue. Nature microbiology. 2019;4(9):1508–15. 10.1038/s41564-019-0476-8 31182801 PMC6784886

[3] Leta S, Beyene TJ, De Clercq EM, Amenu K, Kraemer MUG, Revie CW. Global risk mapping for major diseases transmitted by *Aedes aegypti* and *Aedes albopictus*. International journal of infectious diseases: IJID: official publication of the International Society for Infectious Diseases. 2018;67:25–35. 10.1016/j.ijid.2017.11.026 29196275 PMC5976855

[4] Gould E, Pettersson J, Higgs S, Charrel R, de Lamballerie X. Emerging arboviruses: Why today? One health. 2017;4:1–13. 10.1016/j.onehlt.2017.06.001 28785601 PMC5501887

[5] Bhatt S, Gething PW, Brady OJ, Messina JP, Farlow AW, Moyes CL, et al The global distribution and burden of dengue. Nature. 2013;496(7446):504–7. 10.1038/nature12060 23563266 PMC3651993

[6] UNDP UNDP. A Socio-economic Impact Assessment of the Zika Virus in Latin America and the Caribbean: with a focus on Brazil Colombia and Suriname. 2017.

[7] Wilder-Smith A, Gubler DJ, Weaver SC, Monath TP, Heymann DL, Scott TW. Epidemic arboviral diseases: priorities for research and public health. Lancet Infect Dis. 2017;17(3):e101–e6. 10.1016/S1473-3099(16)30518-7 28011234

[8] Vontas J, Kioulos E, Pavlidi N, Morou E, Della Torre A, Ranson H. Insecticide resistance in the major dengue vectors *Aedes albopictus* and *Aedes aegypti*. Pesticide Biochemistry and Physiology. 2012;104(2):126–31.

[9] Moyes CL, Vontas J, Martins AJ, Ng LC, Koou SY, Dusfour I, et al Contemporary status of insecticide resistance in the major *Aedes* vectors of arboviruses infecting humans. PLoS Negl Trop Dis. 2017;11(7):e0005625 10.1371/journal.pntd.0005625 28727779 PMC5518996

[10] Yakob L, Walker T. Zika virus outbreak in the Americas: the need for novel mosquito control methods. Lancet Glob Health. 2016;4(3):e148–9. 10.1016/S2214-109X(16)00048-6 26848089

[11] Hay RT. SUMO: a history of modification. Molecular cell. 2005;18(1):1–12. 10.1016/j.molcel.2005.03.012 15808504

[12] Liang YC, Lee CC, Yao YL, Lai CC, Schmitz ML, Yang WM. SUMO5, a Novel Poly-SUMO Isoform, Regulates PML Nuclear Bodies. Scientific reports. 2016;6:26509 10.1038/srep26509 27211601 PMC4876461

[13] Rodriguez MS, Dargemont C, Hay RT. SUMO-1 conjugation in vivo requires both a consensus modification motif and nuclear targeting. The Journal of biological chemistry. 2001;276(16):12654–9. 10.1074/jbc.M009476200 11124955

[14] Tatham MH, Jaffray E, Vaughan OA, Desterro JM, Botting CH, Naismith JH, et al Polymeric chains of SUMO-2 and SUMO-3 are conjugated to protein substrates by SAE1/SAE2 and Ubc9. The Journal of biological chemistry. 2001;276(38):35368–74. 10.1074/jbc.M104214200 11451954

[15] Muller S, Hoege C, Pyrowolakis G, Jentsch S. SUMO, ubiquitin's mysterious cousin. Nature reviews Molecular cell biology. 2001;2(3):202–10. 10.1038/35056591 11265250

[16] Vertegaal AC. SUMO chains: polymeric signals. Biochemical Society transactions. 2010;38(Pt 1):46–9. 10.1042/BST0380046 20074033

[17] Wimmer P, Schreiner S. Viral Mimicry to Usurp Ubiquitin and SUMO Host Pathways. Viruses. 2015;7(9):4854–72. 10.3390/v7092849 26343706 PMC4584293

[18] Wilson VG. Sumoylation at the host-pathogen interface. Biomolecules. 2012;2(2):203–27. 10.3390/biom2020203 23795346 PMC3685863

[19] Everett RD, Boutell C, Hale BG. Interplay between viruses and host sumoylation pathways. Nature Reviews Microbiology. 2013;11:400 10.1038/nrmicro3015 23624814

[20] Chiu MW, Shih HM, Yang TH, Yang YL. The type 2 dengue virus envelope protein interacts with small ubiquitin-like modifier-1 (SUMO-1) conjugating enzyme 9 (Ubc9). Journal of biomedical science. 2007;14(3):429–44. 10.1007/s11373-007-9151-9 17265167

[21] Feng T, Deng L, Lu X, Pan W, Wu Q, Dai J. Ubiquitin-conjugating enzyme UBE2J1 negatively modulates interferon pathway and promotes RNA virus infection. Virology Journal. 2018;15:132 10.1186/s12985-018-1040-5 30157886 PMC6114777

[22] Zhu Z, Chu H, Wen L, Yuan S, Chik KK, Yuen TT, et al Targeting SUMO Modification of the Non-Structural Protein 5 of Zika Virus as a Host-Targeting Antiviral Strategy. Int J Mol Sci. 2019;20(2). 10.3390/ijms20020392 30658479 PMC6359730

[23] Su CI, Tseng CH, Yu CY, Lai MMC. SUMO Modification Stabilizes Dengue Virus Nonstructural Protein 5 To Support Virus Replication. J Virol. 2016;90(9):4308–19. 10.1128/JVI.00223-16 26889037 PMC4836324

[24] Choy A, Severo MS, Sun R, Girke T, Gillespie JJ, Pedra JH. Decoding the ubiquitin-mediated pathway of arthropod disease vectors. PloS one. 2013;8(10):e78077 10.1371/journal.pone.0078077 24205097 PMC3804464

[25] Urena E, Pirone L, Chafino S, Perez C, Sutherland JD, Lang V, et al Evolution of SUMO Function and Chain Formation in Insects. Molecular biology and evolution. 2016;33(2):568–84. 10.1093/molbev/msv242 26538142 PMC4866545

[26] Abed M, Bitman-Lotan E, Orian A. The Biology of SUMO-Targeted Ubiquitin Ligases in *Drosophila* Development, Immunity, and Cancer. Journal of developmental biology. 2018;6(1). 10.3390/jdb6010002 PMC5875560 29615551

[27] Matthews BJ, Dudchenko O, Kingan SB, Koren S, Antoshechkin I, Crawford JE, et al Improved reference genome of *Aedes aegypti* informs arbovirus vector control. Nature. 2018;563(7732):501–7. 10.1038/s41586-018-0692-z 30429615 PMC6421076

[28] Su HL, Li SS. Molecular features of human ubiquitin-like SUMO genes and their encoded proteins. Gene. 2002;296(1–2):65–73. 10.1016/s0378-1119(02)00843-0 12383504

[29] Takahashi Y, Toh-e A, Kikuchi Y. A novel factor required for the SUMO1/Smt3 conjugation of yeast septins. Gene. 2001;275(2):223–31. 10.1016/s0378-1119(01)00662-x 11587849

[30] Kotaja N, Karvonen U, Janne OA, Palvimo JJ. PIAS proteins modulate transcription factors by functioning as SUMO-1 ligases. Mol Cell Biol. 2002;22(14):5222–34. 10.1128/mcb.22.14.5222-5234.2002 12077349 PMC139781

[31] Kahyo T, Nishida T, Yasuda H. Involvement of PIAS1 in the sumoylation of tumor suppressor p53. Molecular cell. 2001;8(3):713–8. 10.1016/s1097-2765(01)00349-5 11583632

[32] Truong K, Lee TD, Li B, Chen Y. Sumoylation of SAE2 C Terminus Regulates SAE Nuclear Localization. The Journal of biological chemistry. 2012;287(51):42611–9. 10.1074/jbc.M112.420877 23095757 PMC3522262

[33] Truong K, Lee T, Chen Y. SUMO modification of the E1 Cys domain inhibits its enzymatic activity. Journal of Biological Chemistry. 2012 10.1074/jbc.M112.353789 22403398 PMC3346124

[34] Castillo JC, Robertson AE, Strand MR. Characterization of hemocytes from the mosquitoes *Anopheles gambiae* and *Aedes aegypti*. Insect biochemistry and molecular biology. 2006;36(12):891–903. 10.1016/j.ibmb.2006.08.010 17098164 PMC2757042

[35] Schnettler E, Donald CL, Human S, Watson M, Siu RW, McFarlane M, et al Knockdown of piRNA pathway proteins results in enhanced Semliki Forest virus production in mosquito cells. The Journal of general virology. 2013;94(Pt 7):1680–9. 10.1099/vir.0.053850-0 23559478 PMC3709635

[36] Varjak M, Donald CL, Mottram TJ, Sreenu VB, Merits A, Maringer K, et al Characterization of the Zika virus induced small RNA response in *Aedes aegypti* cells. PLoS Negl Trop Dis. 2017;11(10):e0006010 10.1371/journal.pntd.0006010 29040304 PMC5667879

[37] Dietrich I, Shi X, McFarlane M, Watson M, Blomström A-L, Skelton JK, et al The Antiviral RNAi Response in Vector and Non-vector Cells against Orthobunyaviruses. PLOS Neglected Tropical Diseases. 2017;11(1):e0005272 10.1371/journal.pntd.0005272 28060823 PMC5245901

[38] Harsh S, Ozakman Y, Kitchen SM, Paquin-Proulx D, Nixon DF, Eleftherianos I. Dicer-2 Regulates Resistance and Maintains Homeostasis against Zika Virus Infection in *Drosophila*. Journal of immunology. 2018;201(10):3058–72. 10.4049/jimmunol.1800597 30305326 PMC6219897

[39] Liu Y, Gordesky-Gold B, Leney-Greene M, Weinbren NL, Tudor M, Cherry S. Inflammation-Induced, STING-Dependent Autophagy Restricts Zika Virus Infection in the *Drosophila* Brain. Cell host & microbe. 2018;24(1):57–68 e3. 10.1016/j.chom.2018.05.022 29934091 PMC6173519

[40] Varjak M, Maringer K, Watson M, Sreenu VB, Fredericks AC, Pondeville E, et al *Aedes aegypti* Piwi4 Is a Noncanonical PIWI Protein Involved in Antiviral Responses. mSphere. 2017;2(3). 10.1128/mSphere.00144-17 28497119 PMC5415634

[41] Varjak M, Dietrich I, Sreenu VB, Till BE, Merits A, Kohl A, et al Spindle-E Acts Antivirally Against Alphaviruses in Mosquito Cells. Viruses. 2018;10(2). 10.3390/v10020088 29463033 PMC5850395

[42] Tamberg N, Lulla V, Fragkoudis R, Lulla A, Fazakerley JK, Merits A. Insertion of EGFP into the replicase gene of Semliki Forest virus results in a novel, genetically stable marker virus. The Journal of general virology. 2007;88(Pt 4):1225–30. 10.1099/vir.0.82436-0 17374766 PMC2274952

[43] Mutso M, Saul S, Rausalu K, Susova O, Zusinaite E, Mahalingam S, et al Reverse genetic system, genetically stable reporter viruses and packaged subgenomic replicon based on a Brazilian Zika virus isolate. The Journal of general virology. 2017;98(11):2712–24. 10.1099/jgv.0.000938 29022864

[44] Ruckert C, Ebel GD. How Do Virus-Mosquito Interactions Lead to Viral Emergence? Trends Parasitol. 2018;34(4):310–21. 10.1016/j.pt.2017.12.004 29305089 PMC5879000

[45] Kean J, Rainey SM, McFarlane M, Donald CL, Schnettler E, Kohl A, et al Fighting Arbovirus Transmission: Natural and Engineered Control of Vector Competence in *Aedes* Mosquitoes. Insects. 2015;6(1):236–78. 10.3390/insects6010236 26463078 PMC4553541

[46] Cheng G, Liu Y, Wang P, Xiao X. Mosquito Defense Strategies against Viral Infection. Trends Parasitol. 2016;32(3):177–86. 10.1016/j.pt.2015.09.009 26626596 PMC4767563

[47] Lehembre F, Badenhorst P, Müller S, Travers A, Schweisguth F, Dejean A. Covalent Modification of the Transcriptional Repressor Tramtrack by the Ubiquitin-Related Protein Smt3 in *Drosophila* Flies. Molecular and cellular biology. 2000;20(3):1072–82. 10.1128/mcb.20.3.1072-1082.2000 10629064 PMC85224

[48] Dissanayake SN, Ribeiro JM, Wang MH, Dunn WA, Yan G, James AA, et al. aeGEPUCI: a database of gene expression in the dengue vector mosquito, *Aedes aegypti*. BMC Res Notes. 2010;3:248 10.1186/1756-0500-3-248 20920356 PMC2958886

[49] Etebari K, Hegde S, Saldana MA, Widen SG, Wood TG, Asgari S, et al Global Transcriptome Analysis of *Aedes aegypti* Mosquitoes in Response to Zika Virus Infection. mSphere. 2017;2(6). 10.1128/mSphere.00456-17 29202041 PMC5700376

[50] Huang L, Ohsako S, Tanda S. The lesswright mutation activates Rel-related proteins, leading to overproduction of larval hemocytes in *Drosophila melanogaster*. Dev Biol. 2005;280(2):407–20. 10.1016/j.ydbio.2005.02.006 15882582

[51] Tang X, Li W, Xing J, Sheng X, Zhan W. SUMO and SUMO-Conjugating Enzyme E2 UBC9 Are Involved in White Spot Syndrome Virus Infection in *Fenneropenaeus chinensis*. PloS one. 2016;11(2):e0150324 10.1371/journal.pone.0150324 26927328 PMC4771164

[52] Hu Q, Chen S. Cloning, genomic structure and expression analysis of ubc9 in the course of development in the half-smooth tongue sole (*Cynoglossus semilaevis*). Comparative biochemistry and physiology Part B, Biochemistry & molecular biology. 2013;165(3):181–8. 10.1016/j.cbpb.2013.03.007 23507627

[53] Uhlen M, Fagerberg L, Hallstrom BM, Lindskog C, Oksvold P, Mardinoglu A, et al Proteomics. Tissue-based map of the human proteome. Science. 2015;347(6220):1260419 10.1126/science.1260419 25613900

[54] Uhlen M, Oksvold P, Fagerberg L, Lundberg E, Jonasson K, Forsberg M, et al Towards a knowledge-based Human Protein Atlas. Nature biotechnology. 2010;28(12):1248–50. 10.1038/nbt1210-1248 21139605

[55] Muller S, Matunis MJ, Dejean A. Conjugation with the ubiquitin-related modifier SUMO-1 regulates the partitioning of PML within the nucleus. EMBO J. 1998;17(1):61–70. 10.1093/emboj/17.1.61 9427741 PMC1170358

[56] Mao YS, Zhang B, Spector DL. Biogenesis and function of nuclear bodies. Trends Genet. 2011;27(8):295–306. 10.1016/j.tig.2011.05.006 21680045 PMC3144265

[57] Borden KL. Pondering the promyelocytic leukemia protein (PML) puzzle: possible functions for PML nuclear bodies. Mol Cell Biol. 2002;22(15):5259–69. 10.1128/mcb.22.15.5259-5269.2002 12101223 PMC133952

[58] Borden KL. RING domains: master builders of molecular scaffolds? J Mol Biol. 2000;295(5):1103–12. 10.1006/jmbi.1999.3429 10653689

[59] Brown JR, Conn KL, Wasson P, Charman M, Tong L, Grant K, et al SUMO Ligase Protein Inhibitor of Activated STAT1 (PIAS1) Is a Constituent Promyelocytic Leukemia Nuclear Body Protein That Contributes to the Intrinsic Antiviral Immune Response to Herpes Simplex Virus 1. J Virol. 2016;90(13):5939–52. 10.1128/JVI.00426-16 27099310 PMC4907222

[60] Conn KL, Wasson P, McFarlane S, Tong L, Brown JR, Grant KG, et al Novel Role for Protein Inhibitor of Activated STAT 4 (PIAS4) in the Restriction of Herpes Simplex Virus 1 by the Cellular Intrinsic Antiviral Immune Response. J Virol. 2016;90(9):4807–26. 10.1128/JVI.03055-15 26937035 PMC4836348

[61] Guo J, Chen D, Gao X, Hu X, Zhou Y, Wu C, et al Protein Inhibitor of Activated STAT2 Restricts HCV Replication by Modulating Viral Proteins Degradation. Viruses. 2017;9(10):285 10.3390/v9100285 28973998 PMC5691636

[62] Souza-Neto JA, Sim S, Dimopoulos G. An evolutionary conserved function of the JAK-STAT pathway in anti-dengue defense. Proc Natl Acad Sci U S A. 2009;106(42):17841–6. 10.1073/pnas.0905006106 19805194 PMC2764916

[63] Anglero-Rodriguez YI, MacLeod HJ, Kang S, Carlson JS, Jupatanakul N, Dimopoulos G. *Aedes aegypti* Molecular Responses to Zika Virus: Modulation of Infection by the Toll and Jak/Stat Immune Pathways and Virus Host Factors. Front Microbiol. 2017;8:2050 10.3389/fmicb.2017.02050 29109710 PMC5660061

[64] Jupatanakul N, Sim S, Anglero-Rodriguez YI, Souza-Neto J, Das S, Poti KE, et al Engineered *Aedes aegypti* JAK/STAT Pathway-Mediated Immunity to Dengue Virus. PLoS Negl Trop Dis. 2017;11(1):e0005187 10.1371/journal.pntd.0005187 28081143 PMC5230736

[65] Salazar MI, Richardson JH, Sanchez-Vargas I, Olson KE, Beaty BJ. Dengue virus type 2: replication and tropisms in orally infected *Aedes aegypti* mosquitoes. BMC Microbiol. 2007;7:9 10.1186/1471-2180-7-9 17263893 PMC1797809

[66] Parikh GR, Oliver JD, Bartholomay LC. A haemocyte tropism for an arbovirus. The Journal of general virology. 2009;90(Pt 2):292–6. 10.1099/vir.0.005116-0 19141437

[67] Carissimo G, Pondeville E, McFarlane M, Dietrich I, Mitri C, Bischoff E, et al Antiviral immunity of *Anopheles gambiae* is highly compartmentalized, with distinct roles for RNA interference and gut microbiota. Proc Natl Acad Sci U S A. 2015;112(2):E176–85. 10.1073/pnas.1412984112 25548172 PMC4299212

[68] Bhaskar V, Smith M, Courey AJ. Conjugation of Smt3 to Dorsal May Potentiate the *Drosophila* Immune Response. Molecular and cellular biology. 2002;22(2):492–504. 10.1128/mcb.22.2.492-504.2002 11756545 PMC139748

[69] Handu M, Kaduskar B, Ravindranathan R, Soory A, Giri R, Elango VB, et al SUMO-Enriched Proteome for *Drosophila* Innate Immune Response. G3 (Bethesda, Md). 2015;5(10):2137–54. 10.1534/g3.115.020958 26290570 PMC4592996

[70] Dostert C, Jouanguy E, Irving P, Troxler L, Galiana-Arnoux D, Hetru C, et al The Jak-STAT signaling pathway is required but not sufficient for the antiviral response of *drosophila*. Nat Immunol. 2005;6(9):946–53. 10.1038/ni1237 16086017

[71] Zhang R, Zhu Y, Pang X, Xiao X, Zhang R, Cheng G. Regulation of Antimicrobial Peptides in *Aedes aegypti* Aag2 Cells. Front Cell Infect Microbiol. 2017;7:22 10.3389/fcimb.2017.00022 28217557 PMC5291090

[72] Varma MG, Pudney M. The growth and serial passage of cell lines from *Aedes aegypti* (L.) larvae in different media. Journal of medical entomology. 1969;6(4):432–9. 10.1093/jmedent/6.4.432 5360492

[73] Peleg J. Growth of arboviruses in monolayers from subcultured mosquito embryo cells. Virology. 1968;35(4):617–9. 10.1016/0042-6822(68)90293-6 5677803

[74] Rodriguez-Andres J, Rani S, Varjak M, Chase-Topping ME, Beck MH, Ferguson MC, et al Phenoloxidase activity acts as a mosquito innate immune response against infection with Semliki Forest virus. PLoS Pathog. 2012;8(11):e1002977 10.1371/journal.ppat.1002977 23144608 PMC3493465

[75] Watret GE, Pringle CR, Elliott RM. Synthesis of bunyavirus-specific proteins in a continuous cell line (XTC-2) derived from *Xenopus laevis*. The Journal of general virology. 1985;66 (Pt 3):473–82. 3973561 10.1099/0022-1317-66-3-473

[76] Royle J, Donald CL, Merits A, Kohl A, Varjak M. Differential effects of lipid biosynthesis inhibitors on Zika and Semliki Forest viruses. Veterinary journal (London, England: 1997). 2017;230:62–4. 10.1016/j.tvjl.2017.10.009 29102599 PMC5726355

[77] Livak KJ, Schmittgen TD. Analysis of relative gene expression data using real-time quantitative PCR and the 2(-Delta Delta C(T)) Method. Methods. 2001;25(4):402–8. 10.1006/meth.2001.1262 11846609

[78] Taylor SC, Nadeau K, Abbasi M, Lachance C, Nguyen M, Fenrich J. The Ultimate qPCR Experiment: Producing Publication Quality, Reproducible Data the First Time. Trends Biotechnol. 2019;37(7):761–74. 10.1016/j.tibtech.2018.12.002 30654913

[79] Shevchenko A, Tomas H, Havlis J, Olsen JV, Mann M. In-gel digestion for mass spectrometric characterization of proteins and proteomes. Nature protocols. 2006;1(6):2856–60. 10.1038/nprot.2006.468 17406544

[80] Cox J, Mann M. MaxQuant enables high peptide identification rates, individualized p.p.b.-range mass accuracies and proteome-wide protein quantification. Nature biotechnology. 2008;26(12):1367–72. 10.1038/nbt.1511 19029910

[81] Matic I, van Hagen M, Schimmel J, Macek B, Ogg SC, Tatham MH, et al In vivo identification of human small ubiquitin-like modifier polymerization sites by high accuracy mass spectrometry and an in vitro to in vivo strategy. Mol Cell Proteomics. 2008;7(1):132–44. 10.1074/mcp.M700173-MCP200 17938407 PMC3840926

[82] Notredame C, Higgins DG, Heringa J. T-Coffee: A novel method for fast and accurate multiple sequence alignment. Journal of molecular biology. 2000;302(1):205–17. 10.1006/jmbi.2000.4042 10964570

[83] Pettersen EF, Goddard TD, Huang CC, Couch GS, Greenblatt DM, Meng EC, et al UCSF Chimera—a visualization system for exploratory research and analysis. Journal of computational chemistry. 2004;25(13):1605–12. 10.1002/jcc.20084 15264254

[84] Kelley LA, Sternberg MJ. Protein structure prediction on the Web: a case study using the Phyre server. Nature protocols. 2009;4(3):363–71. 10.1038/nprot.2009.2 19247286

[85] Xu Y, Plechanovova A, Simpson P, Marchant J, Leidecker O, Kraatz S, et al Structural insight into SUMO chain recognition and manipulation by the ubiquitin ligase RNF4. Nat Commun. 2014;5:4217 10.1038/ncomms5217 24969970 PMC4083429

