# Supplementary figures and images for: The SUMOylation pathway suppresses arbovirus replication in Aedes aegypti cells
Source: PLoS Pathog. 2020 Dec 22;16(12):e1009134. doi: 10.1371/journal.ppat.1009134 (PMC7802965; doi:10.1371/journal.ppat.1009134)

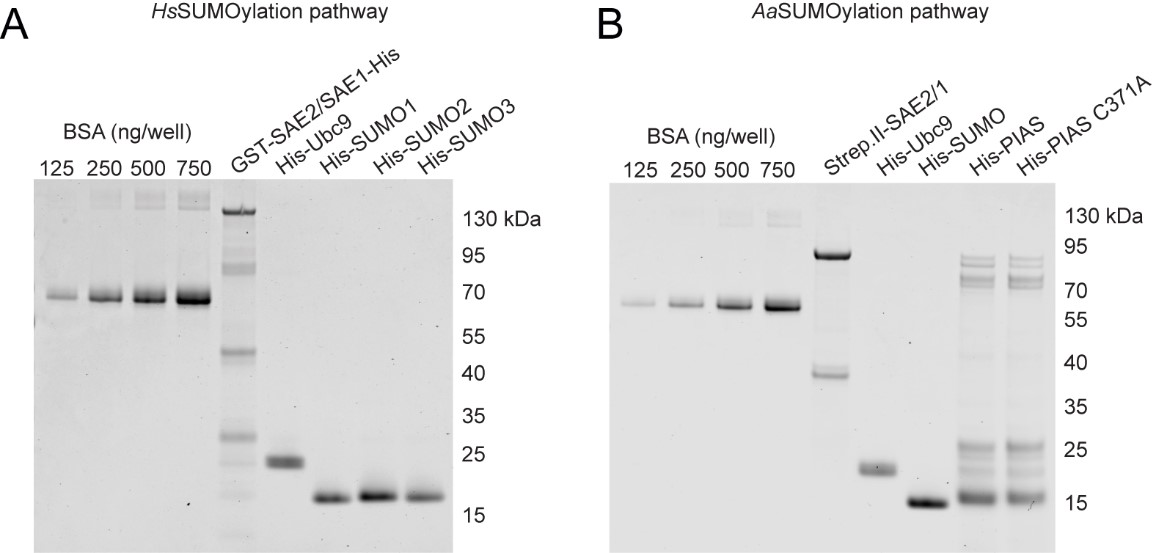

Supplement: S1 Fig — 6xHis- or Strep.II-tagged proteins of the (A) H. sapiens and (B) Ae. aegypti SUMOylation pathways were expressed in bacteria and purified through Nickle- or Biotin-affinity chromatography. Samples were resolved by SDS-PAGE and Coomassie stained. A BSA gradient is included to assess concentration. Molecular weights indicated. (JPG) [file ppat.1009134.s001.jpg]

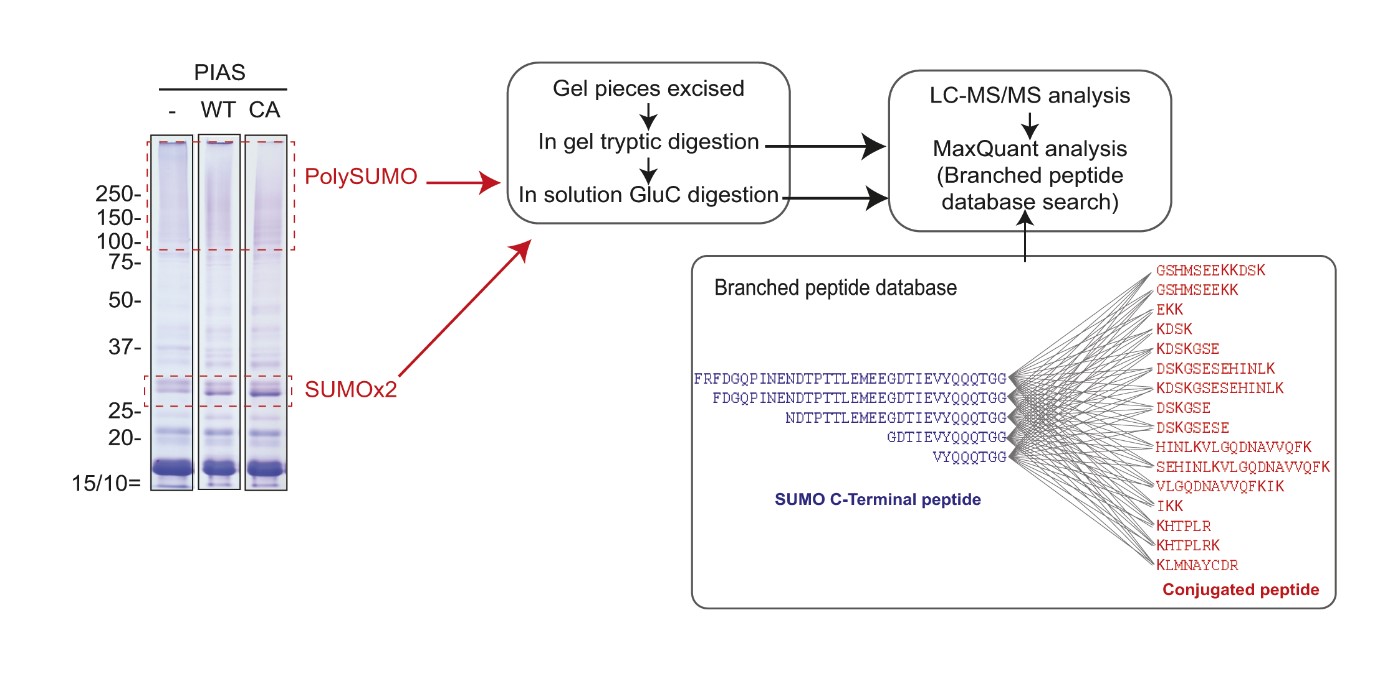

Supplement: S2 Fig — Coomassie gel showing in vitro conjugation assay products from reactions either lacking AaPIAS (-), or including wild-type (WT) or inactive C371A mutant variants (CA). Schematic explaining sample processing and mass spectrometry analysis using a branched peptide database is shown. (JPG) [file ppat.1009134.s002.jpg]

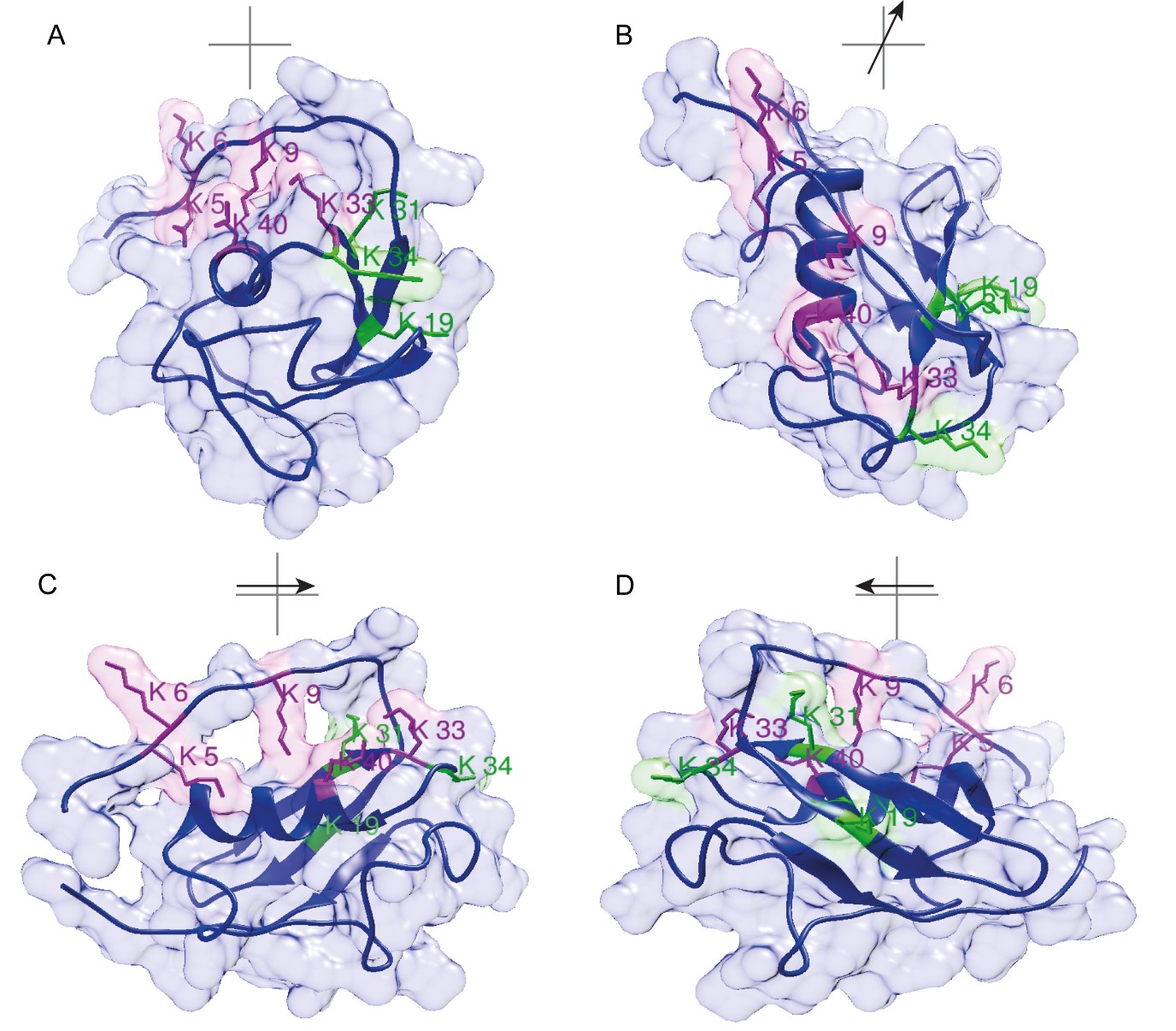

Supplement: S3 Fig — SUMO internal lysine residues identified as being prominent acceptors of SUMO modification in Fig 3 were plotted on the predicted structure of AaSUMO (blue). Green indicates poor acceptors of SUMO modification (total branched peptide intensity <3x10^9^), while magenta indicates the lysine residues which are predominantly modified (total branched peptide intensity >3x10^9^). K19, K31, and K34 are shown in green, while K5, K6, K9, K33, and K40 are in magenta. (A) Front view. (B) Top view. (C, D) side view rotated ± 90° from A. (JPG) [file ppat.1009134.s003.jpg]

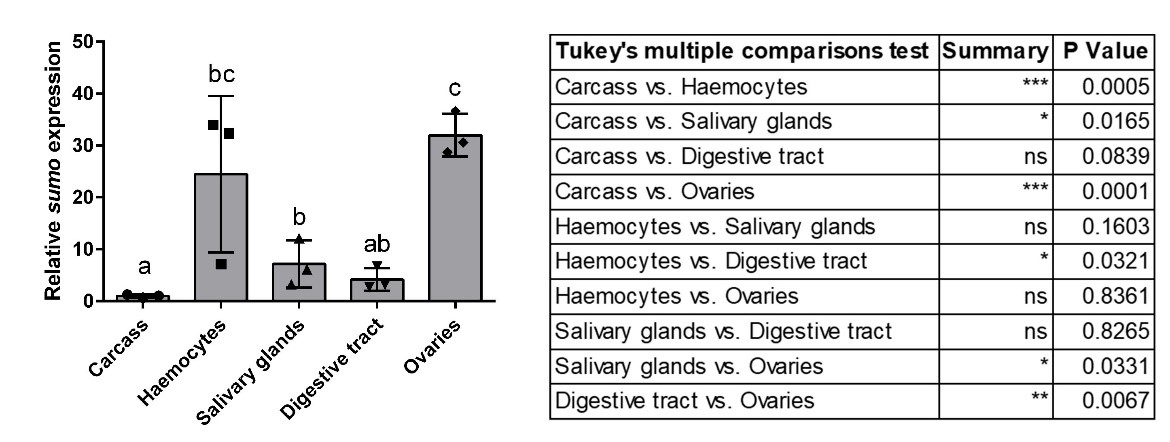

Supplement: S4 Fig — Data presented on Fig 4A were subjected to an ANOVA test (F4,10 = 17.99, p = 0.0001) followed by a Tukey’s post hoc multiple comparison test. Letters above bars indicate post hoc significance. Groups with the same letter are not significantly different. Table shows p values for each group comparison. (JPG) [file ppat.1009134.s004.jpg]

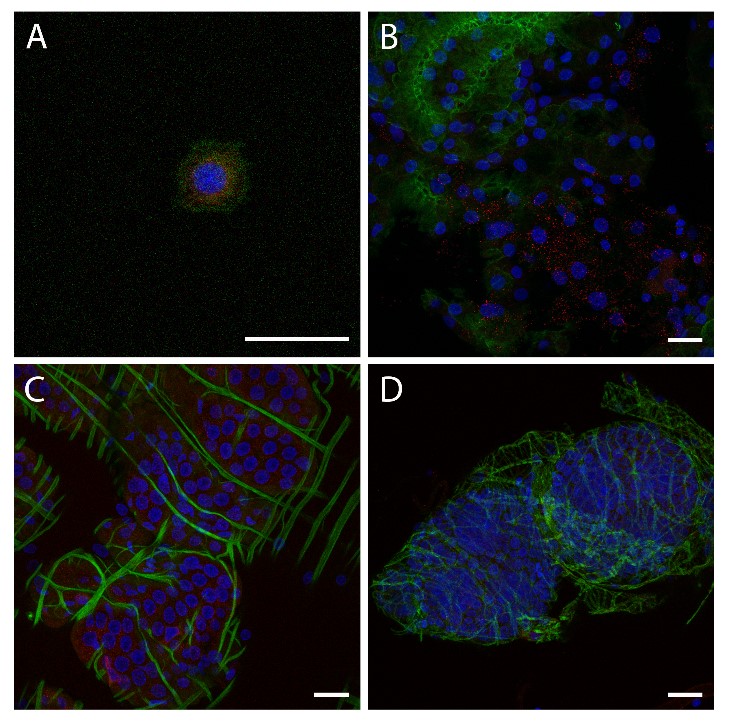

Supplement: S5 Fig — Immunofluorescence assay without primary antibody on perfused haemocytes (A), salivary glands (B), midgut (C) and ovaries (D). The signal was revealed by an Alexa Fluor 568 goat anti-mouse IgG (H+L) diluted 1:1000 (red). Nuclei are stained by DAPI (blue) and F-actin is stained by Phalloidin 488 (green). The images were acquired on a Zeiss LSM 710 inverted confocal microscope with 40X or 63X oil-immersion objective and using the same parameters as those used for samples incubated with anti-SUMO primary antibody (Fig 5). Scale bar is 20 μm. (JPG) [file ppat.1009134.s005.jpg]

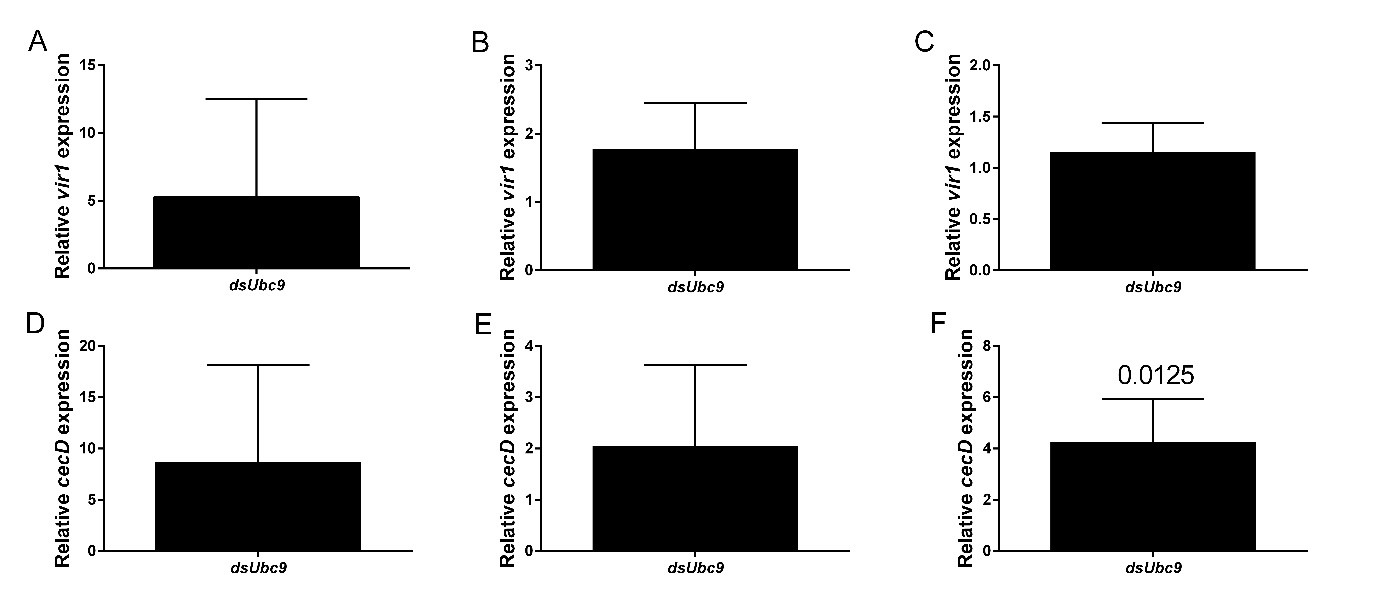

Supplement: S6 Fig — Expression of vir1 and cecropin D (cecD) was analysed in dsUbc9- and dsLacZ-treated cells (as described in Fig 5) by RT-qPCR. N = 5 independent biological replicates. Values normalized to ribosomal S7 and expressed relative to dsLacZ-treated control samples set to 1; RQ mean and SD plotted. Statistical analysis, one sample (two-tailed) t test to a hypothetical mean of 1 (dsLacZ control), significant probability (P) values (≤ 0.05) shown. (A, B, C) Expression of vir1 in BUNV-, SFV- and ZIKV-infected cells, respectively. (D, E, F) Expression of cecropin D in BUNV-, SFV- and ZIKV-infected cells, respectively. (JPG) [file ppat.1009134.s006.jpg]

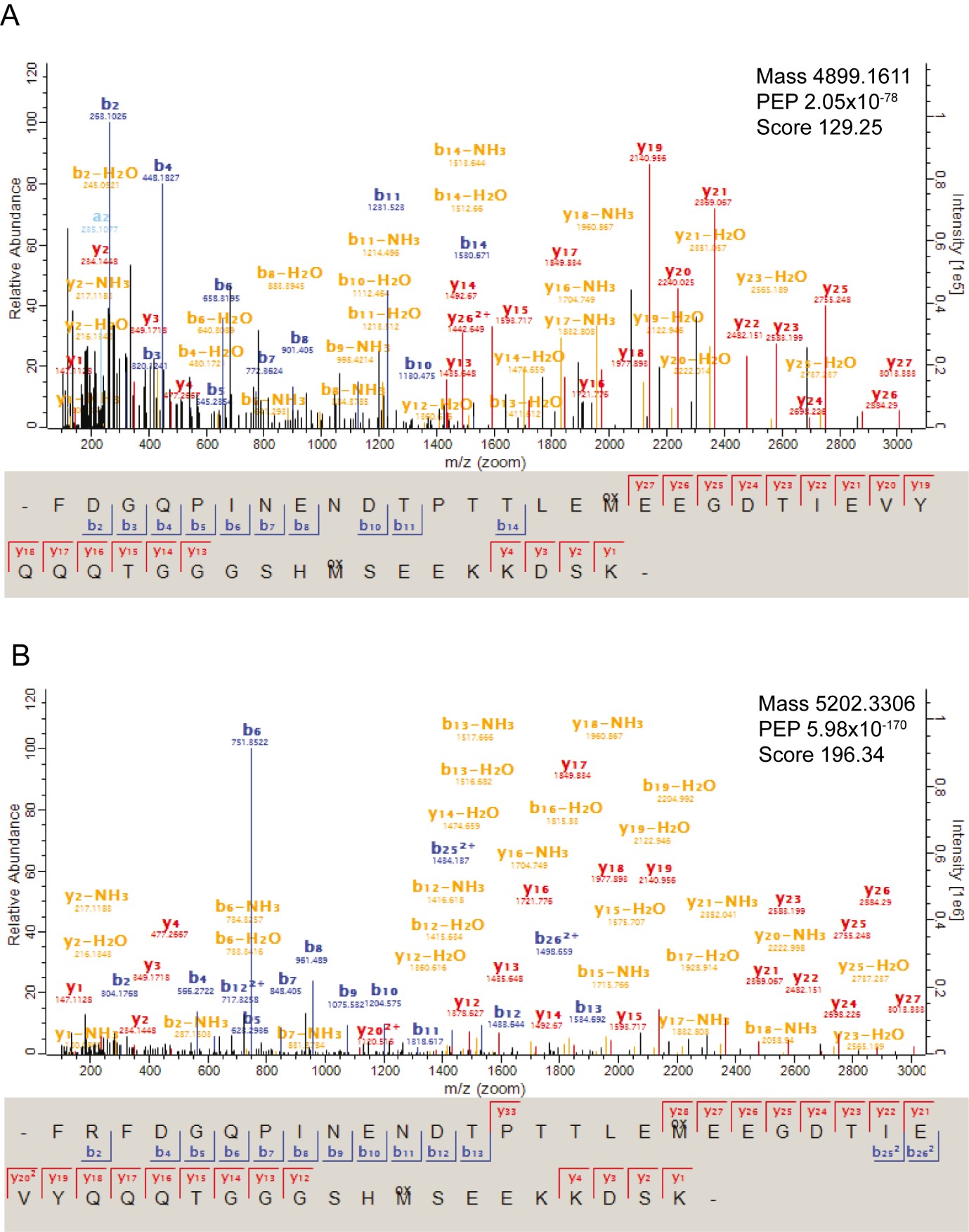

Supplement: S7 Fig — (A, B) Best spectra for two different peptides providing evidence for polymerisation of AaSUMO via lysine 5. See S2 File for details. Note, due to the use of a concatenated database of N-terminal fusions of the SUMO C-terminus to potential substrate peptides, many fragments are not annotated. These are mostly b series ions from the substrate peptide up to the modified lysine. (JPG) [file ppat.1009134.s007.jpg]

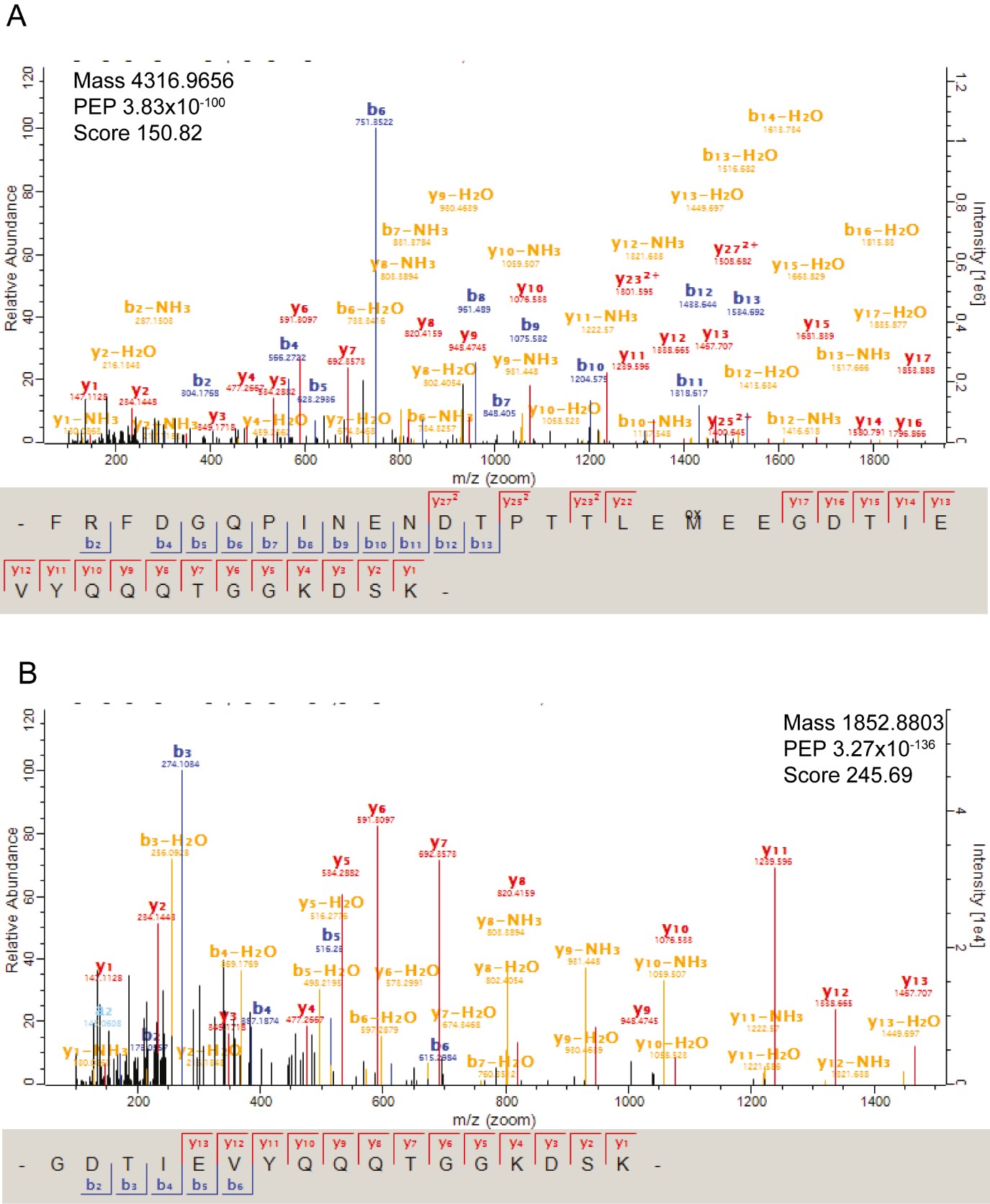

Supplement: S8 Fig — (A, B) Best spectra for two different peptides providing evidence for polymerisation of AaSUMO via lysine 6. See S2 File for details. Note, due to the use of a concatenated database of N-terminal fusions of the SUMO C-terminus to potential substrate peptides, many fragments are not annotated. These are mostly b series ions from the substrate peptide up to the modified lysine. (JPG) [file ppat.1009134.s008.jpg]

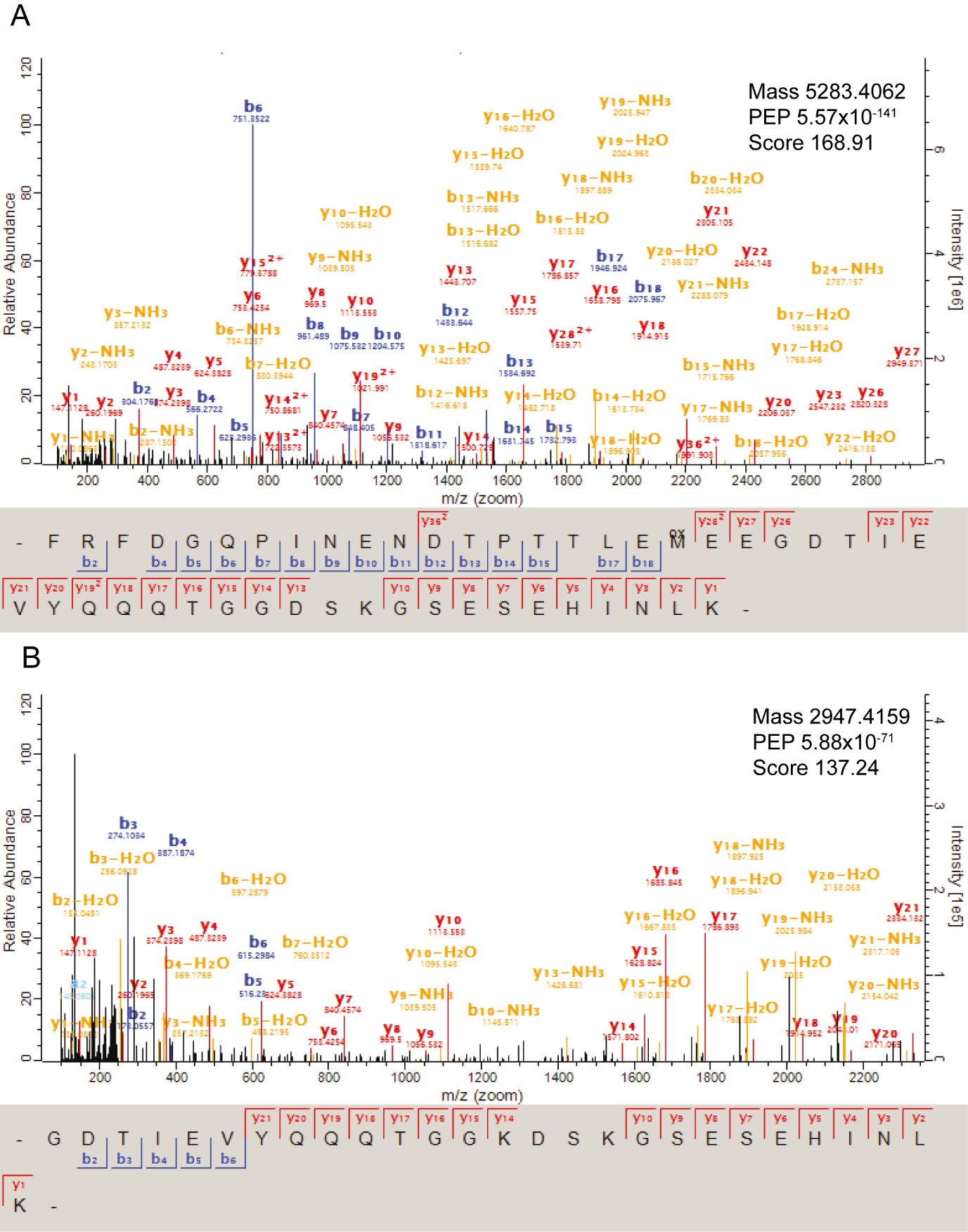

Supplement: S9 Fig — (A, B) Best spectra for two different peptides providing evidence for polymerisation of AaSUMO via lysine 9. See S2 File for details. Note, due to the use of a concatenated database of N-terminal fusions of the SUMO C-terminus to potential substrate peptides, many fragments are not annotated. These are mostly b series ions from the substrate peptide up to the modified lysine. (JPG) [file ppat.1009134.s009.jpg]

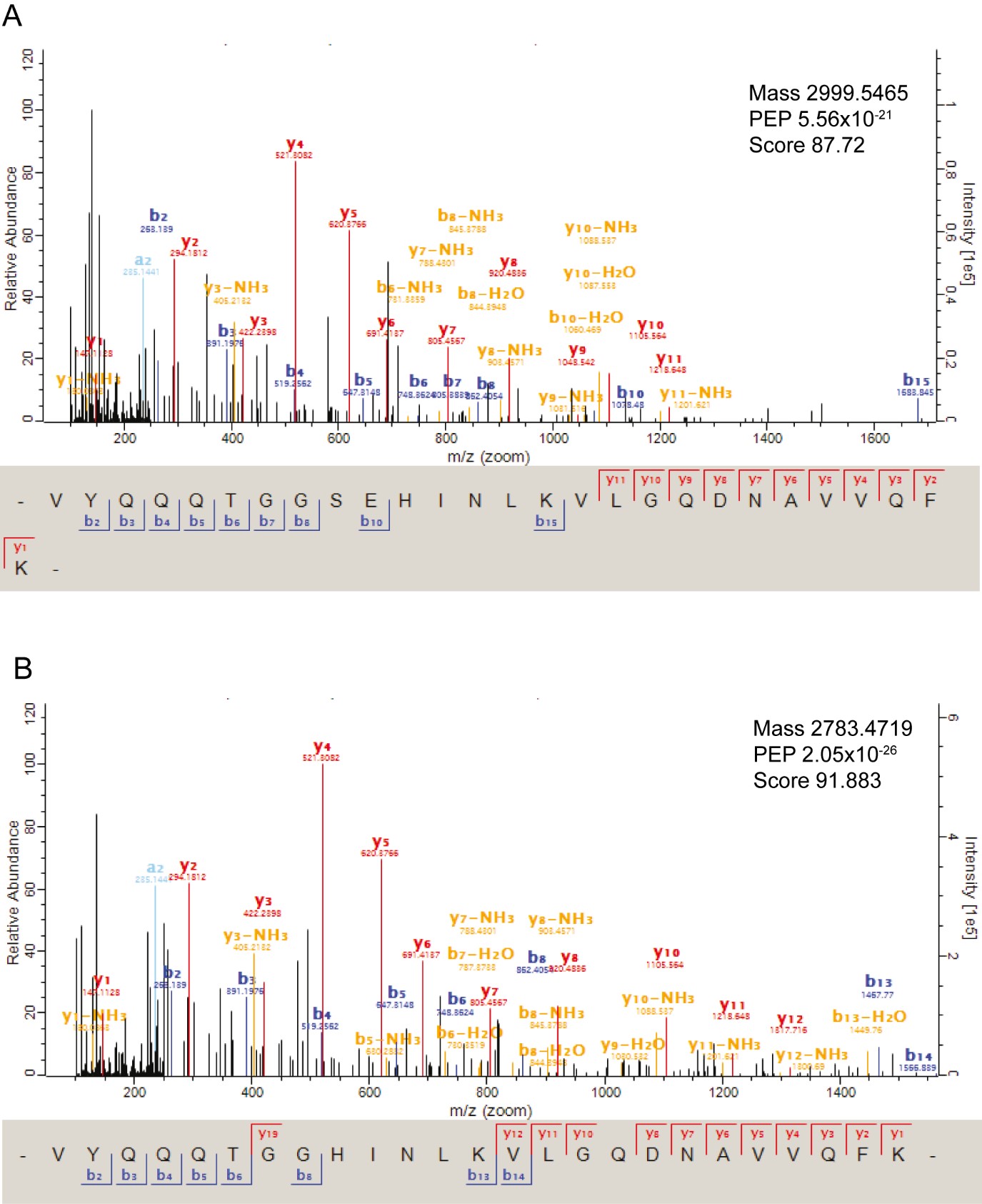

Supplement: S10 Fig — (A, B) Best spectra for two different peptides providing evidence for polymerisation of AaSUMO via lysine 19. See S2 File for details. Note, due to the use of a concatenated database of N-terminal fusions of the SUMO C-terminus to potential substrate peptides, many fragments are not annotated. These are mostly b series ions from the substrate peptide up to the modified lysine. (JPG) [file ppat.1009134.s010.jpg]

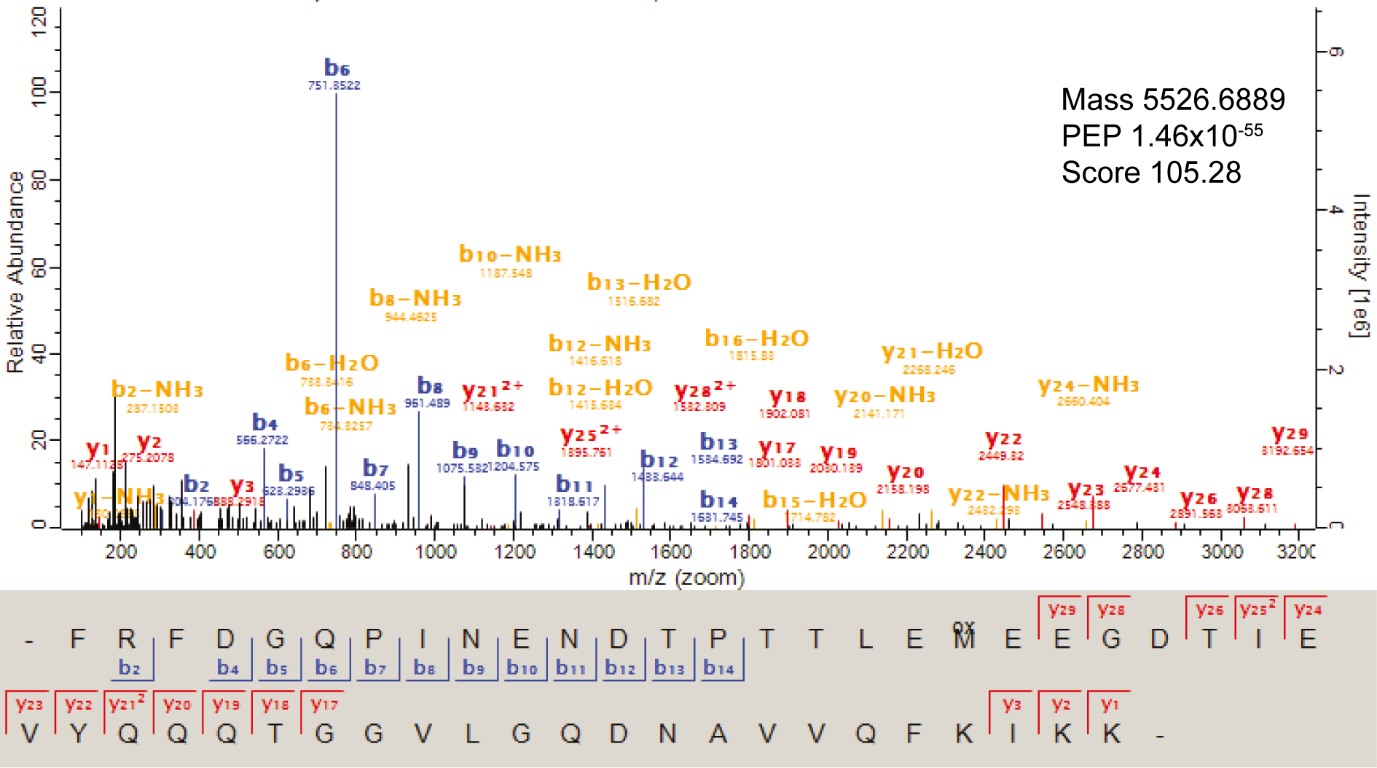

Supplement: S11 Fig — Best spectrum for the only peptide providing evidence for polymerisation of AaSUMO via lysine 31. See S2 File for details. Note, due to the use of a concatenated database of N-terminal fusions of the SUMO C-terminus to potential substrate peptides, many fragments are not annotated. These are mostly b series ions from the substrate peptide up to the modified lysine. (JPG) [file ppat.1009134.s011.jpg]

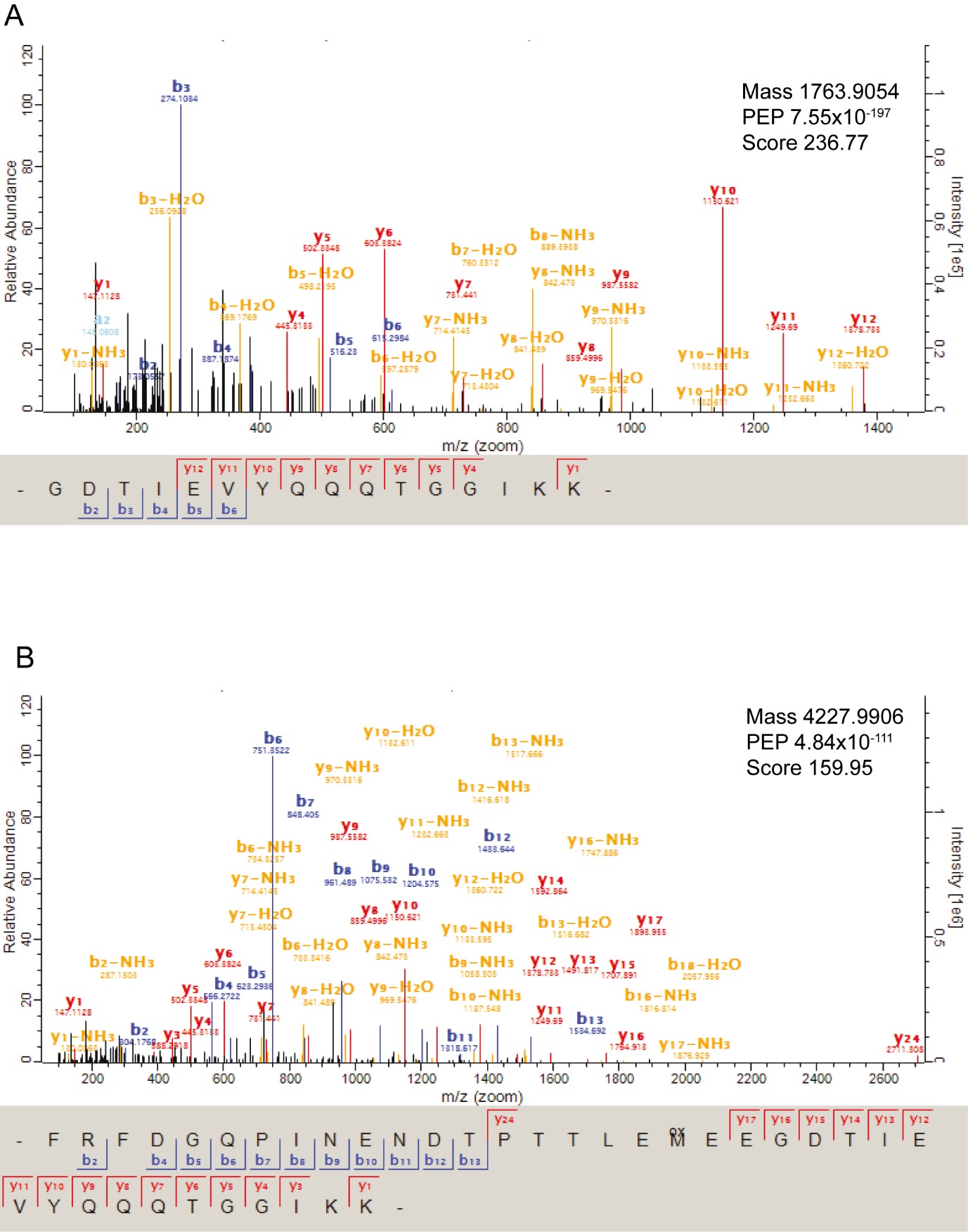

Supplement: S12 Fig — (A, B) Best spectra for two different peptides providing evidence for polymerisation of AaSUMO via lysine 33. See S2 File for details. Note, due to the use of a concatenated database of N-terminal fusions of the SUMO C-terminus to potential substrate peptides, many fragments are not annotated. These are mostly b series ions from the substrate peptide up to the modified lysine. (JPG) [file ppat.1009134.s012.jpg]

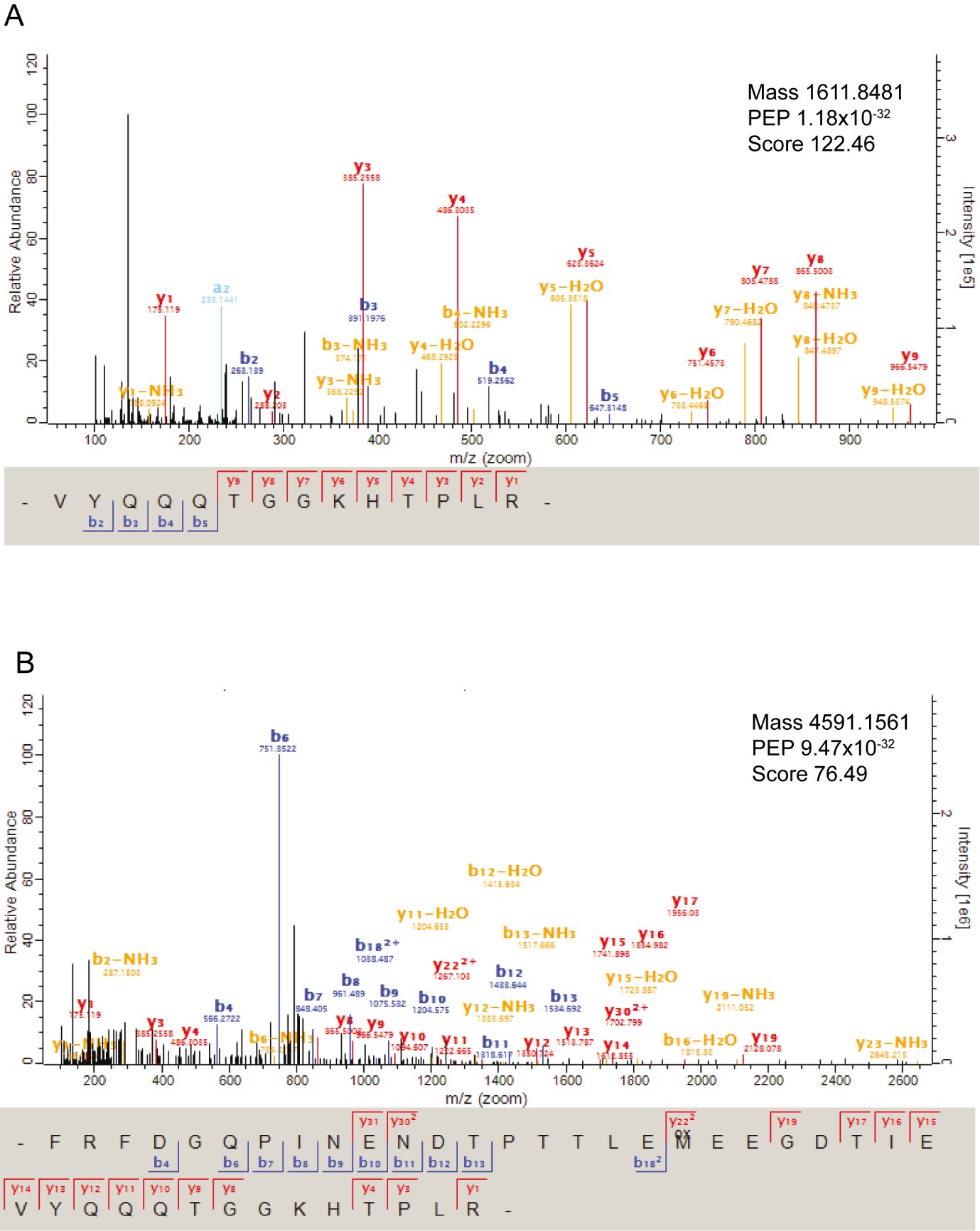

Supplement: S13 Fig — (A, B) Best spectra for two different peptides providing evidence for polymerisation of AaSUMO via lysine 34. See S2 File for details. Note, due to the use of a concatenated database of N-terminal fusions of the SUMO C-terminus to potential substrate peptides, many fragments are not annotated. These are mostly b series ions from the substrate peptide up to the modified lysine. (JPG) [file ppat.1009134.s013.jpg]

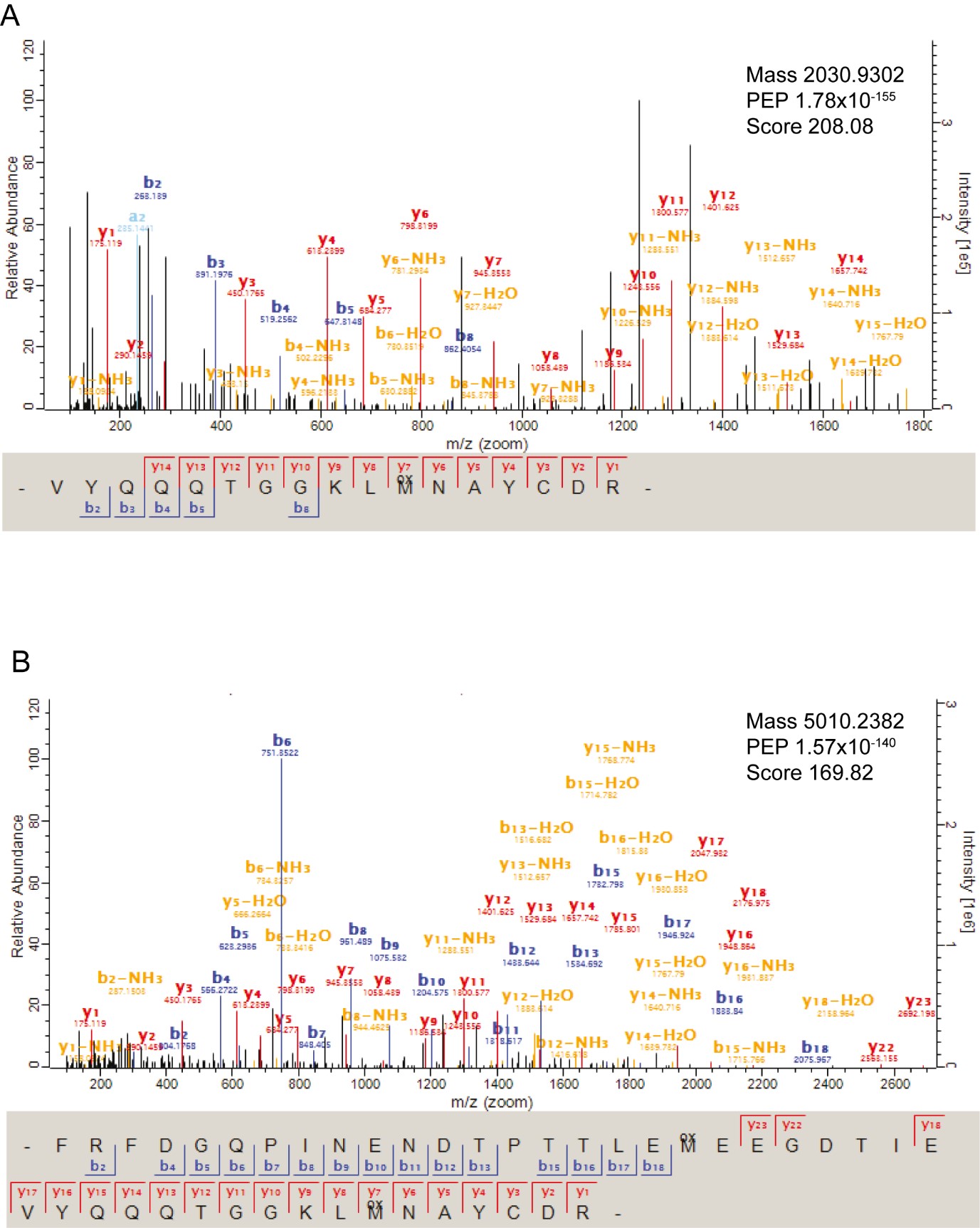

Supplement: S14 Fig — (A, B) Best spectra for two different peptides providing evidence for polymerisation of AaSUMO via lysine 40. See S2 File for details. Note, due to the use of a concatenated database of N-terminal fusions of the SUMO C-terminus to potential substrate peptides, many fragments are not annotated. These are mostly b series ions from the substrate peptide up to the modified lysine. (JPG) [file ppat.1009134.s014.jpg]
