# Supplementary material for: The SUMOylation pathway suppresses arbovirus replication in Aedes aegypti cells
Source: PLoS Pathog. 2020 Dec 22;16(12):e1009134. doi: 10.1371/journal.ppat.1009134 (PMC7802965; doi:10.1371/journal.ppat.1009134)
Supplement: S1 Table — (DOCX) [file ppat.1009134.s015.docx]

**S1 Table. Primer sequences.**

| **Name** | **Use** | **Sequence (5’-3’)** |
| --- | --- | --- |
| dsRNA *Aa*SUMO F | dsRNA | taatacgactcactatagggTCCGAATCGGAGCACATTAA |
| dsRNA *Aa*SUMO R | dsRNA | taatacgactcactatagggCATGTTCCATGGCTTGTACG |
| dsRNA *Aa*Ubc9 F | dsRNA | taatacgactcactatagggATGTCCGGAATTGCGATCGC |
| dsRNA *Aa*Ubc9 R | dsRNA | taatacgactcactatagggGGATCCTTGATGTTCGGTTCGT |
| dsRNA *Aa*PIAS F | dsRNA | taatacgactcactatagggAAcAGGAGGATTACTTCCCACC |
| dsRNA *Aa*PIAS R | dsRNA | taatacgactcactatagggGCAGACGGGACAGTTCCAAG |
| dsRNA *Aa*Ago2 F | dsRNA | gtaatacgactcactatagggGCCCTCAACAAGAAACACC |
| dsRNA *Aa*Ago2 R | dsRNA | gtaatacgactcactatagggGGCGTTGATCTTGAGCCA |
| dsRNA *Aa*LacZ F | dsRNA | taatacgactcactatagggGTCGCCAGCGGCACCGCGCCTTTC |
| dsRNA *Aa*LacZ R | dsRNA | taatacgactcactatagggCCGGTAGCCAGCGCGGATCATCGG |
| Q_PCR *Aa*SUMO F | qPCR | CGCCAATTTTCAGCACAC |
| Q_PCR *Aa*SUMO R | qPCR | CGGATCCCTTCGAGTCC |
| Q_PCR *Aa*Ubc9 F | qPCR | GGACTGGCGTCCGGCGATC |
| Q_PCR *Aa*Ubc9 R | qPCR | TTACTCAGTGGCAGCCATGGCCC |
| Q_PCR *Aa*PIAS F | qPCR | CGGCCGATTACACACG |
| Q_PCR *Aa*PIAS R | qPCR | GACAATCTTGTTATCGATGGCT |
| Q_PCR S7 F | qPCR | CCAGGCTATCCTGGAGTTG |
| Q_PCR S7 R | qPCR | GACGTGCTTGCCGGAGAAC |
| Q_PCR LacZ F | qPCR | CCGGCTGTGCCGAAAT |
| Q_PCR LacZ R | qPCR | GCGGCTGATGTTGAACTGG |
| Q_PCR Ago2 F | qPCR | GAGCAAACAAATATCCCA |
| Q_PCR Ago2 R | qPCR | TGGTGTCGCTTTTGGAC |
| Q_ZIKV NS3 F | qPCR | ATCTGTATGGAGGTGGGTGC |
| Q_ZIKV NS3 R | qPCR | CTCTCCCTCAATGGCTGCTA |
| Q_SFV Stru F | qPCR | GCCGAAAACGCAGCCCAAG |
| Q_SFV Stru R | qPCR | GGCGTACCCAGTGACCTTTCCTT |
| Q_BUNV M F | qPCR | CGGAATTCAGTAGTGTACTACC |
| Q_BUNV M R | qPCR | GACATATGCTTGATTGAAGCAAGCATG |
| Vir1 QFOR | qPCR | CCATCGGATGACACGGAGTA |
| Vir1 QREV | qPCR | GTGTCCACAATGCCATCGAA |
| CecD QFOR | qPCR | CGCTTTGGTCCTGCTAGGT |
| CecD QREV | qPCR | AAGCCTTGAATACTCGCTTGC |
| *Aa*SAE2 F | Cloning | GCACTGCAGGAAACAATAGC |
| *Aa*SAE2 R | Cloning | CTCAGCTCCCGCTTCCGGATCC |
| *Aa*SAE2 F1 | Cloning | TCTCCGGATACTGCGGATCC |
| *Aa*SAE2 R1 | Cloning | GAATGATGTTTCCCGCCATGG |
| *Aa*SAE2 F2 | Cloning | GCTTTGAAGTCAAATCCATGG |
| *Aa*SAE2 R2 | Cloning | CAATGTGTACTCTTACC |
| *Aa*SAE1 F | Cloning | ACCGCAGACGCCGCCTTG |
| *Aa*SAE1 R | Cloning | GATATTAAAGTATATACG |
| *Aa*SAE1 F1 | Cloning | GAACGAACCGTGCGGAAGCTTC |
| *Aa*SAE1 R1 | Cloning | CTGAGCCCGAGACAAAGAAGCTTC |
| pET28-*Aa*SUMO R | Cloning | TATCCTCGAGTTATCCGCCTGTCTG |
| pET28-*Aa*SUMO F | Cloning | GGCGCATATGTCTGAAGAAAAAAAGG |
| pET28-SUMO chimera F | Cloning | ATTGCATATGTCCGAGGAGAAGCC |
| *Aa*PIAS F | Cloning | CCGGACCGGTATGAGAAAAACGCGG |
| *Aa*PIAS R | Cloning | CGCGGCGGCCGCTCATATCTTGG |
| pET-*Aa*Ubc9 F | Cloning | CCGGACCGGTTCCGGAATTGCGATCGC |
| pET-*Aa*Ubc9 R | Cloning | CGCGCTCGAGCTACTCCGTGGCAGCCATGG |

Lower case nucleotides represent T7 promoter sequence.
